# Developing Creep and Stress Relaxation Models to Assess the Service Life of an Additive Manufactured Industrial-Scale Recuperator Utilizing Inconel 625 and AISI 310S Materials

**DOI:** 10.3390/ma16227226

**Published:** 2023-11-18

**Authors:** Christos Papalexis, Dimitrios Rakopoulos, Nikolaos Nikolopoulos, Alessandro Della Rocca, Guido Jochler, Oriana Tassa, Christos Kalligeros, Panteleimon Tzouganakis, Vasilios Spitas

**Affiliations:** 1 Centre for Research & Technology Hellas, Chemical Process & Energy Resources Institute, 52, Egialias Str., 15125 Athens, Greece; rakopoulos@certh.gr (D.R.); n.nikolopoulos@certh.gr (N.N.); ptzouganakis@uniwa.gr (P.T.); 2 Laboratory of Machine Design, National Technical University of Athens, 9 Iroon Polytechniou, 15780 Zografou, Greece; ckalligeros@mail.ntua.gr (C.K.); vspitas@mail.ntua.gr (V.S.); 3 Global R&D, Tenova SpA, 16153 Genova, Italy; alessandro.dellarocca@tenova.com; 4 Rina Consulting Centro Sviluppo Materiali SpA, 00128 Rome, Italy; guido.jochler@rina.org (G.J.); oriana.tassa@rina.org (O.T.)

**Keywords:** creep and stress relaxation model, additive manufactured recuperator, Inconel 625, Stainless Steel 310, creep failure criterion

## Abstract

This work is focused on the development of creep and stress relaxation models on Inconel 625 and Stainless Steel 310 materials for additive manufacturing. At the end, the operational lifespan of an industrial-scale additive manufactured recuperator is evaluated. An industrial-scale recuperator for burners with a highly complex geometry is manufactured using Continuous Wave SLM and Pulsed Wave Selective Laser Melting techniques. The recuperator operates under steady but high thermal loads, reaching temperatures of up to 875 °C. Therefore, its service life is assessed, considering creep and stress relaxation phenomena. Two different materials are evaluated: Inconel 625 and Stainless Steel 310. Tensile testing has been conducted on samples at various temperatures to acquire material parameters, incorporating appropriately the anisotropic nature of the materials. Creep parameters were determined through creep experiments and data from the literature, and the recuperator response was simulated by FEA modelling. Analytical creep and stress relaxation models were proposed based on the simulation results for each material to predict their creep response. The service life was determined by applying a custom failure criterion based on the creep testing data. The Inconel 625 recuperator exhibits a service life that is significantly higher compared to any burner’s life, while the Stainless Steel 310 recuperator exhibits approximately 27 years of service life. Both materials are considered suitable; however, Inconel 625 offers higher resistance to creep according to creep tests, and due to its lower thermal expansion coefficient, the resulting thermal stresses are lower.

## 1. Introduction

Recuperators play a crucial role in enhancing the overall efficiency of burners by recovering waste heat from flue gases and preheating incoming air or fuel. By doing so, they significantly reduce fuel consumption and emissions, making them indispensable for sustainable and cost-effective operations [1].

However, despite their importance, recuperators face challenges related to long-term reliability and durability under high-temperature conditions. Creep and stress relaxation are two significant issues that can affect their performance over time [2]. Creep refers to the gradual deformation of materials when subjected to constant high-stress levels at elevated temperatures [3]. Stress relaxation occurs when materials experience a reduction in stress over time while maintaining a constant strain [4].

Additive manufacturing (AM) methods enable the fabrication of structural components with complex geometries and unique mechanical properties. Hence, it presents a potential solution, allowing advanced designs suitable for installation in various burner systems with improved resistance to creep and stress relaxation [5,6], thus promising the necessary reliability for industrial use. This approach is expected to revolutionize recuperator manufacturing and performance, contributing to more sustainable and reliable industrial processes.

The development of AM begun for polymer materials [7]. However, progress has been made in the fabrication of metallic parts by AM techniques to serve various needs in the industry [7,8,9]. One of the most common AM technologies for printing metal materials is Selective Laser Melting (SLM), which uses powder and laser fusion in a layer-wise manner to fabricate metallic parts with complex geometries [10].

Inconel is a nickel-based superalloy with exceptional thermomechanical performance. Kanagarajah et al. [11] studied the microstructure and the mechanical properties of Inconel 939 through SLM. Its mechanical properties were better than those of Inconel processed by casting, but anisotropic. Trosch et al. [12] mention that the mechanical properties of SLM-fabricated Inconel 718 samples are highly dependent on layer orientation. However, their strength was higher than their casted and forged counterparts. Yadroitsev et al. [13] described the macrostructural texture of Inconel 625 manufactured through SLM. The strength of AM samples exceeds that of wrought samples. In addition, the level of its anisotropy seems to decrease at elevated temperatures [14]. Despite this opportunity, limited data are available about the mechanical properties of AM Inconel 625 and its level of anisotropy [15]. Even more insufficient are the data in the literature about the mechanical properties of AM AISI 310S. AISI 316 stainless steel is a widely used material in industry similar to AISI 310S. AM-fabricated 316 steel has better strength properties than the conventionally produced AISI 316 steel [16].

The present study examines Inconel 625 and AISI 310S in the context of a highly complex geometric recuperator for burners, produced through Selective Laser Melting (SLM), under high thermal loads. Tensile testing is conducted on AM-fabricated samples, and material parameters are acquired for both materials at various temperatures. The study captures the anisotropic nature of the materials through experimental data and incorporates it into the Finite Element Analysis (FEA) model. Creep parameters are determined through creep experiments and data in the literature, differentiated based on temperature thresholds. A custom failure criterion is employed based on creep testing data to determine the service life of the recuperator. This work contributes to the material characterization of AM-fabricated Inconel 625 and 316 Stainless Steel, for which there are currently limited data in the literature regarding creep properties. The study also provides valuable insights into the creep and stress relaxation response of an industrial-scale case study, demonstrating the reliability of AM-fabricated parts for industrial applications.

## 2. Materials and Methods

### 2.1. Material Description

There are two alternatives for the material of the recuperator: Inconel 625 and AISI 310 stainless steel. Inconel 625 is a nickel-based superalloy that is highly resistant to corrosion and high temperatures. Industries that use Inconel include chemical/power processing, aerospace and automotive, seawater or offshore oil and gas, and recuperators [17,18]. Similarly, parts made by AISI 310S show a high level of corrosion resistance, toughness, and ductility, as well as a high resistance to acids that can be used for various applications. The chemical composition of the materials can be found in Table 1 and Table 2.

The recuperator for this study will be produced not through a conventional manufacturing processes, but through 3D printing. Consequently, one concern that could arise would be if the Inconel 625 or AISI 310S materials maintain their excellent mechanical properties in high-temperature environments even when produced through 3D printing. Son et al. [19] performed high-temperature creep tests of additively manufactured (AM) Inconel 625 and wrought Inconel 625 at 650 °C and 800 °C over a stress range of 65 MPa to 658 MPa. The 3D-printed Inconel 625 showed an equal or even higher creep strength than wrought (manufactured) Inconel 625 for all heat treatments. Furthermore, the tensile strength of the 3D-printed Inconel 625 alloy was almost the same as that of conventional wrought or cast Inconel 625 alloys at room temperature and 760 °C. Similarly, in the case of stainless steel, the strength properties of SLM-produced stainless steel are higher than the corresponding properties after conventional manufacturing processes such as rolling. Special mention is made for the obtained yield strengths, which are significantly higher than those for wrought products while maintaining high elongation values [20]. Consequently, not only do 3D-printed Inconel 625 and AISI 310S maintain their properties regarding their strength in high-temperature environments, but in some cases, they exhibit even better behavior than wrought materials.

Inconel 625 and AISI 310S materials will be investigated for the AM fabrication of a recuperator using both Continuous Wave and Pulsed Wave SLM strategies. Thus, samples were produced through both techniques for every material.

Since the recuperator is manufactured with SLM AM techniques, the material will not be isotropic. For that reason, tensile tests were conducted on samples built in horizontal (x and y) and vertical (z) directions at different temperatures. From the tensile tests, the stress–strain curves were obtained from tensile tests on horizontally oriented samples at room temperature and from tensile tests on vertically oriented samples at 22 °C, 600 °C, 750 °C, 900 °C, and 1050 °C. All stress–strain curves acquired experimentally are presented in Appendix A.

From the tensile test data, it was observed that the values of Y_S_ and the UTS are not significantly different for the two-layer orientations, and the Y_S_ and the UTS are considered equal to the lowest value in every direction. On the contrary, concerning the elastic region, the anisotropic behavior of the material was taken into consideration at every temperature through linear interpolation to the given values of E. The density and thermal expansion coefficient of the materials were obtained from the ANSYS Workbench (ANSYS, Canonsburg, Pennsylvania, USA) material database as a function of the temperature. The orthotropic elasticity models of every material imported in the simulations are presented analytically in the Appendix A.

Considering that the recuperator is manufactured vertically (in the axial direction) in SLM while the hatching was rotated by 67° between scanned layers, the lower value of E is attributed to the axial direction, while the greater value of E is attributed to the other two directions depending on the appropriate element orientation.

### 2.2. FEA Model Set Up

**Figure 1:**
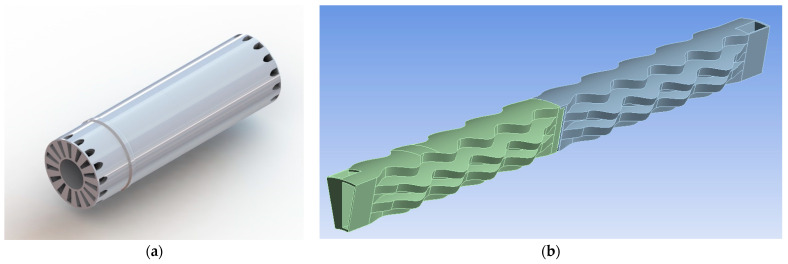
Geometry: (**a**) whole recuperator model, (**b**) studied CAD model.

The simulated structural model is only a section of the whole recuperator to reduce the computational cost. More specifically, the geometry (Figure 1a) is considered axisymmetric, and the simulated section represents 1/15 of the whole geometry (Figure 1b). The CAD model has been defeatured (mainly by the removal of fillets) to allow high-quality mesh to be generated.

**Figure 2:**
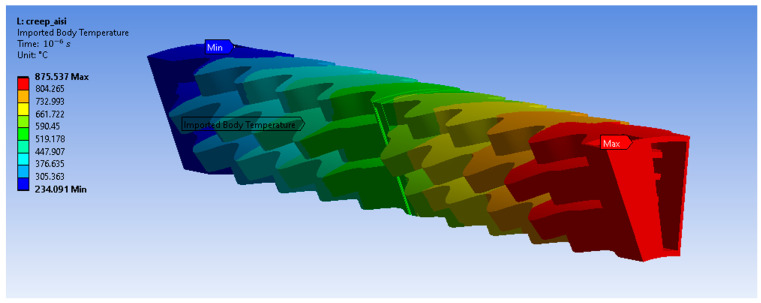
Temperature distribution along the recuperator.

The loads applied to the recuperator are due to the imported non-uniform temperature distribution (Figure 2) and the pressure of the air and exhaust gas under steady-state operation.

**Figure 3:**
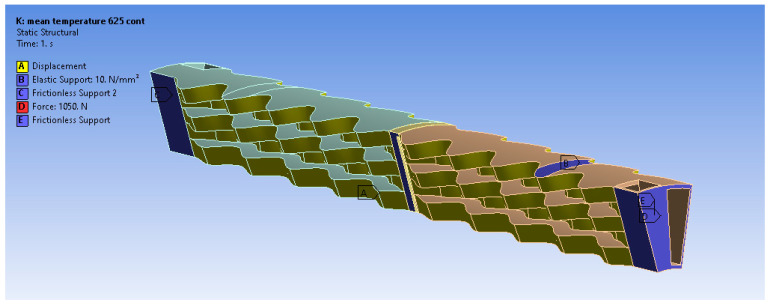
Model’s boundary conditions.

The recuperator is mounted on the burner body by two supports, which lie in the colder part of its body. The front surface—denoted by the letter E (Figure 3)—is welded to a flange. The second support—denoted by the letter Β—is located peripherally to the recuperator, and it attaches the recuperator to the outer flange. In steady state, due to conduction, the temperature of the flanges and the temperature of the recuperator in that region are homogenized. However, since the thermal expansion coefficient is not the same for the recuperator and the flanges, they are not expanding at the same rate as the temperature increases. In order to model this phenomenon, a shear force and an elastic support are applied to the first and the second support, respectively. Considering that the flanges are rigid, the magnitudes of the force and the support stiffness are dependent only on the thermal expansion coefficients of the flange and the recuperator. Their values are such that the resulting radial deformation at the supports differs by ${\delta \varepsilon }_{r}$ (Equation (1)) compared to the resulting radial deformation when the thermal expansion coefficients are the same.

(1) $${\delta \varepsilon }_{r}=(a_{2}-a_{1})\Delta T$$

where

$a_{1}$ is the thermal expansion coefficient of the material of the recuperator at room temperature.
$a_{2}$ is the thermal expansion coefficient of the material of the flanges at room temperature, which is considered $a_{2}=12.4\;{\times \;10}^{-6}\;C^{-1}$, which is the thermal expansion coefficient of typical steel according to ANSYS material database.
ΔT is the temperature rise at the cross section, which is equal to ΔT=212 °C at the roller support and ΔT=300 °C at the radial support.

The circumferential deformation is fixed to zero at the side surfaces of the studied sector in order to satisfy the assumption of the axisymmetric nature of the recuperator.

**Figure 4:**
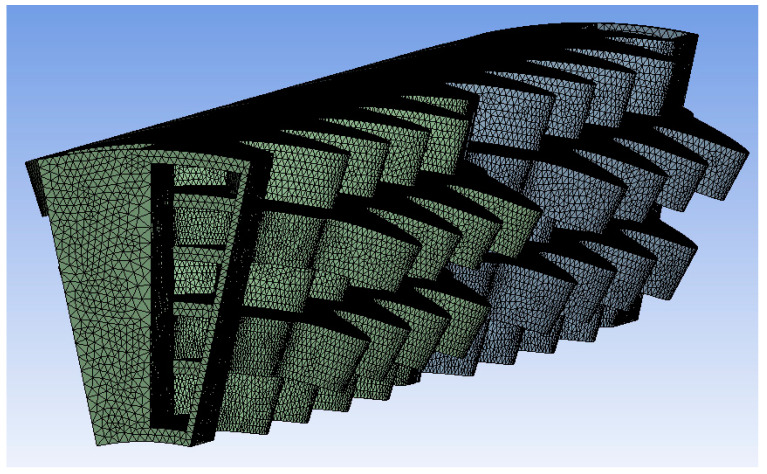
Tetrahedral mesh.

**Figure 5:**
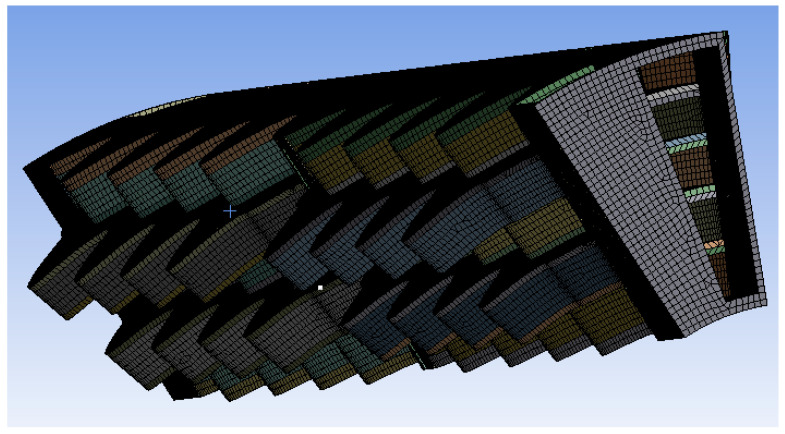
Hexahedral dominant mesh.

The mesh strategy aims at adequately capturing the bending of the recuperator walls; two meshing strategies were implemented, one using tetrahedral elements (1,647,873 elements) and one using hex-dominant elements (364,566 elements), in order to ensure mesh independence. The final mesh is presented in Figure 4 and Figure 5, respectively.

### 2.3. Creep Models

#### 2.3.1. Creep and Stress Relaxation

When a metal structure is subjected to stress or strain over a period of time, the amount of plastic strain at the end of the time period will be greater than it was at the first moment of loading. This gradual increase in the plastic strain is caused by two different mechanisms: creep and stress relaxation. Creep is the tendency of a solid material to deform under the influence of constant mechanical stresses. It occurs due to continuous exposure to stresses and generally increases with higher temperatures, stress amplitude, and exposure time [3]. The strain of the material undergoing creep could be described by the following function:

(2) $$\varepsilon =f\left(\sigma ,\;T,\;t\right)$$

**Figure 6:**
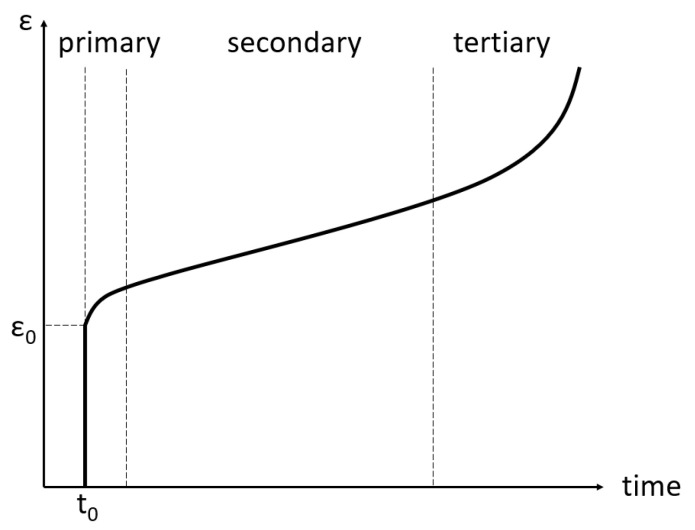
Strain (ε) due to constant stress over an extended period of time.

The behaviour of creep can be divided into three stages, as presented in Figure 6. In the primary stage, the strain rate decreases exponentially over time until it reaches a constant value. This constant strain rate defines the secondary stage or steady-state creep. Finally, in the tertiary creep stage, the strain rate exponentially increases, eventually leading to failure.

Many models of the creep phenomenon have been presented in the literature. These models are focused on the secondary stage of creep, in which the strain rate is constant. The purpose of the models is to determine the constant strain rate of the secondary stage of the creep in order to predict the time in which the material will fail. The secondary stage of creep occupies the majority of the creep phenomenon; therefore, an adequate approximation of the rapture time could be obtained from the secondary-stage creep model.

The most common way of modelling the secondary creep stage is based on Equation (3):

(3) $${\dot{\varepsilon }}_{s}=\sigma^{n}f\left(T\right)$$

where σ is the applied stress and n is an exponent based on the material. In general, the exponent n is constant within a range of stress and temperature. It should be noted that the equivalent strain rate of the secondary stage of creep is significantly higher when the temperature is above $T_{m}/2,$ compared to the temperature being below $\;T_{m}/2$. Therefore, in order to simulate the secondary stage of creep with higher level of accuracy, two different models (the exponent n and the temperature relation  f(T)) should be obtained for each case (above and below $T_{m}/2$). The temperature dependence on the creep f(T) is obtained from an Arhenius-type expression since creep is a thermally activated process [21,22]. Therefore,

(4) $${f(T)=e}^{-\frac{Q_{c}}{RT}}$$

where $Q_{c}$ is the apparent activation energy, R is the gas constant, and T is the temperature. As a consequence, from Equations (3) and (4), the strain rate, ${\dot{\varepsilon }}_{s},$ could be obtained from Equation (5) (Norton Law)

(5) $${\dot{\varepsilon }}_{s}=A\sigma^{n}e^{-\frac{Q_{c}}{RT}}$$

where A is a constant that depends on the material. In addition, various other models have been presented in the literature by researchers where the shear modulus and Young’s modulus (Equations (6)–(8)) have been included in the strain rate relation [21,23].

(6) $${\dot{\varepsilon }}_{s}=A^{\prime }D{\left(\frac{\sigma }{E}\right)}^{n}e^{-\frac{Q_{c}}{RT}}$$

(7) $${\dot{\varepsilon }}_{s}=A^{\prime \prime }D{\left(\frac{\sigma }{E}\right)}^{n}$$

(8) $${\dot{\varepsilon }}_{s}=A^{\prime \prime \prime }\frac{DGb}{kT}{\left(\frac{\sigma }{E}\right)}^{n}$$

where D is the self-diffusivity, b is the Burgers vector, and k is the Boltzmann constant.

Since it is difficult to obtain the aforementioned parameters for a specific material, it is equivalent to express the secondary creep in the following general form:

(9) $${\dot{\varepsilon }}_{s}=c_{1}\sigma^{c_{2}}e^{-\frac{c_{3}}{T}}$$

In order to accurately evaluate the correct constants $,c_{1}$, $c_{2}$ and $c_{3\;},$ creep experiments were conducted, and the appropriate constants were selected so that the creep response obtained from Equation (9) will be more in agreement with the observed experimental strain rates.

**Figure 7:**
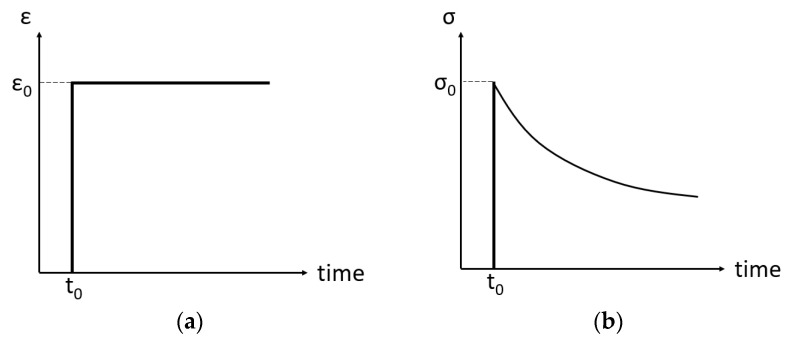
Strain (**a**) and stress (**b**) response over time due to stress relaxation.

Contrarily to creep, stress relaxation is defined as the gradual reduction in stress with time at a constant strain. In practice, the strain is increased up to a preset value and held, after which the stress reduction can be monitored. During relaxation, a portion of stored elastic energy is converted to plastic strain, leading to a relaxation of the applied stress with time. Along with the reduction in stresses, the mounting reactions of the structure are also relieved. Consequently, stress relaxation has a similar effect as cold springing, though it requires a longer period of time. The stress reduction over time is presented in Figure 7. The stress relaxation depends on many different factors, mainly time, temperature, and stress level.

Since stress relaxation is a phenomenon that is most apparent and dominant in polymers, there are a lot of models that aim to simulate the effect of stress relaxation through viscoelastic material models. The most commonly used viscoelastic models are the Maxwell and Voight models for the prediction of stress relaxation and creep, respectively.

Generally, the models use to predict the effect of stress relaxation can differ for different materials. In order to provide a global model for stress relaxation, Obukhov [4] suggested a power-law dependency, described by Equation (10).

(10) $$\sigma \left(t\right)=\frac{\sigma_{0}}{1-\left[1-\left(t/t^{*}\right)\left(1^{1-n}\right)\right]}$$

where *σ*_0_ is the maximum stress at the time of loading removal (t*), and *n* is a material parameter.

Junisbekov et al. [24] suggested a different model using a power series, focusing on the stress relaxation in polyamides (Equation (11)).

(11) $$\sigma \left(t\right)=\sum_{m,n}A_{m,n}{\left[\ln\left(1+t\right)\right]}^{m}{\left(\varepsilon_{0}^{\prime }\right)}^{n}$$

More models can be found in the literature attempting to predict the stress relaxation behavior of certain materials. Their main shortcoming is that no model available currently can accurately describe the stress relaxation behavior globally for any material, or even for a group of materials with similar properties. In addition, apart from the material itself, different conditions can also affect the accuracy of these models, including the initial loading (magnitude and speed), the temperature, the loading medium, the presence of friction and wear, and their long-term storage conditions. However, due to the similarities between creep and stress relaxation, scientists are often trying to predict the effect of stress relaxation using parameters and data from creep models or even using the creep models themselves.

Even though creep and stress relaxation are two distinct phenomena, published studies show that their effects can be predicted using similar models for each one of them [25,26,27]. The experimental results show that similar variation trends have been observed regarding creep and stress relaxation behavior [28]. Especially as the temperature increases, the correspondence between the creep and stress relaxation results becomes higher until they are fairly similar at higher temperatures [25]. This similar behavior allows scientists to calculate the necessary material parameters for stress relaxation models from creep tests and vice versa [29,30]. Through rigorous testing, a creep model derived from the material’s creep data has been established as a standard modelling technique for the estimation of both creep and stress relaxation responses for the majority of materials [26]. Actually, many textbooks that discuss creep and stress relaxation assume, at least tacitly, that the two are complementary [25]. Consequently, a creep model fitted with experimental data for a specific material can predict the effect of stress relaxation on the same material with sufficient accuracy. Therefore, only creep material parameters are used to predict the recuperator’s response and failure under both creep and stress relaxation phenomena.

#### 2.3.2. Creep Models of AM-Fabricated Materials

**Figure 8:**
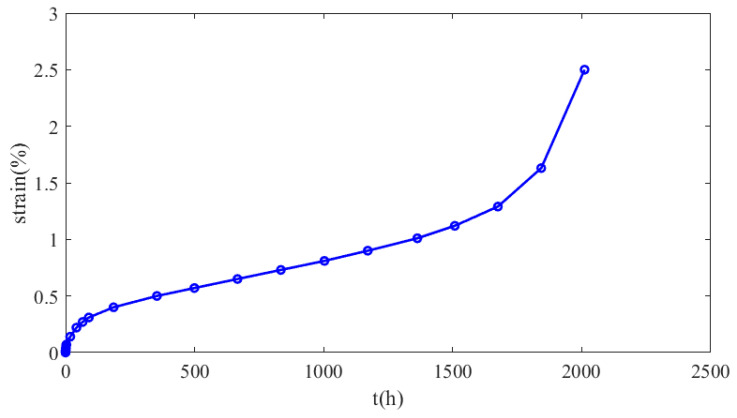
Creep curve of sample 5.

**Figure 9:**
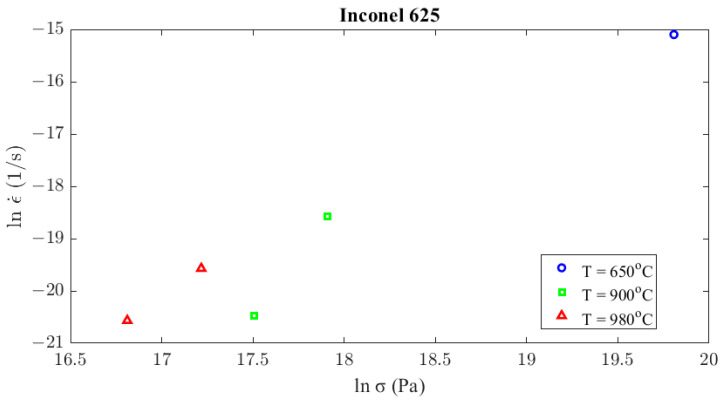
Secondary creep strain rate calculated from creep experiments according to stress and temperature conditions.

To define the creep model governing the AΜ Inconel 625 material, creep tests were conducted on the samples under various temperature and loading conditions. Each creep test was terminated after a rupture occurred, and the duration of each test is mentioned in Table 3, along with the experimental conditions and the resulting strain rate. The strain rate refers to the slope of the secondary stage of the experimental curves. Figure 8 displays the creep curve of sample 5 indicatively. The values of the parameters *c*_1_, *c*_2_, and *c*_3_ of Equation (9) must be determined as a function of temperature using the experimental data presented in Figure 9.

Frost and Ashby [31] reported that the value of Nickel activation energy is 170 kJ/mol. Moore et al. [32] found different values of self-diffusion activation energy for the Inconel 625 alloy depending on the temperature. The value of the activation energy obtained was 108.3 ± 6.6 kJ/mol above 700 °C and 46.6 ± 12.2 kJ/mol below 650 °C. Between 650 and 700 °C, a significantly higher value of the activation energy equal to 527.7 ± 23.1 kJ/mol was found. The activation energy value found from de Oliveira et al. [33] was 407 kJ/mol. Son et al. [19] calculated that the creep activation energy of the AM-produced Inconel 625 is 273 kJ/mol, and the creep activation energy of wrought Inconel 625 is 284 kJ/mol. The calibrated value for the reference wrought Inconel 625, as indicated by Graca et al. [34], is 275 kJ/mol, and it is very close to that of the AM-produced Inconel 625. The activation energy for the current study was taken to be equal to 27 5 kJ/mol. Hence, the $c_{3}$ parameter is given by Equation (12).

(12) $$c_{3}=\frac{Q_{c}}{R}=33,000\;K$$

As presented, the creep behavior is different at temperatures below and above a critical one, which is about the half of its melting point $T_{m}$. The value of Inconel 625’s critical temperature is given by Equation (13).

(13) $$T_{crit}^{Inconel}=\frac{T_{m}^{Inconel}}{2}\approx 680\;{}^{\circ}C$$

Son et al. [19] performed creep tests on Inconel 625 and found that the value of the stress exponent ($c_{2}$) at 650 °C is 10.8 and at 800 °C is 6.8. Using the experimental results of sample 1, which was tested under 650 °C, and since $c_{3}$ is already determined by Equation (12) and $c_{2}$ was obtained by [19], the parameter $c_{1}$ is calculated at that temperature by Equation (14) and the result is presented in Equation (14).

(14) $$c_{1}\left(923\;K\right)={5.37\times 10}^{-85}\;for\;T=923\;K<T_{m}/2$$

Above the critical temperature, four different creep tests were conducted. From the experimental values, the *c*_2_ and *c*_1_ parameters will be determined as a function of the temperature above the critical one. From Equation (9) and the experimental results in Table 3, the stress exponent at 900 °C and 980 °C is calculated:

(15) $$\begin{array}{c} c_{2}\left(1173\;K\right)=4.69\\ c_{2}(1253\;K)=2.46 \end{array}$$

**Figure 10:**
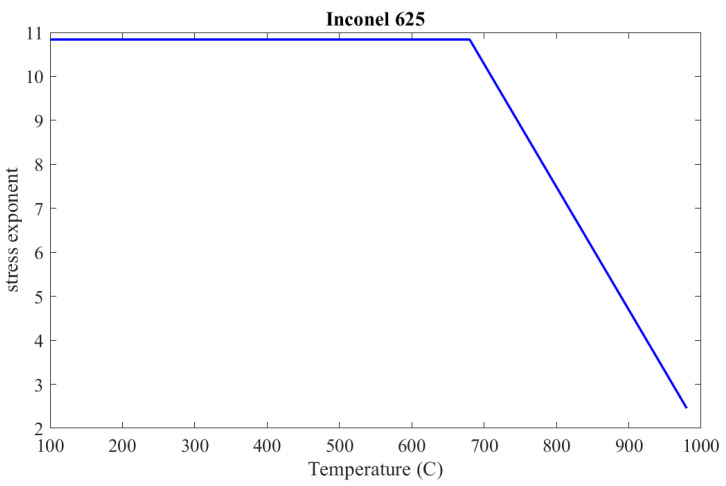
Inconel 625 stress exponent, *c*_2_, as a function of temperature.

From the results in (15) and assuming that the stress exponent is decreasing linearly as the temperature increases [35], in the range from the critical temperature to 980 °C, the stress exponent, as a continuous function of temperature, can be determined according to Formula (16), which is illustrated in Figure 10.

(16) $$c_{2}\left(T\right)=\left\{\begin{array}{c} 10.84,\;\;\;\;T<953\;K\\ -0.028\;T+37.47,\;\;\;\;953\;K\leq T<1253\;K \end{array}\right.$$

The above values of the stress exponent have a good correlation with the literature [19].

The *c*_1_ parameter at 900 °C and 980 °C is obtained:

(17) $$\begin{array}{c} c_{1}\left(1173\;K\right)=4.554\;{\times 10}^{-33}\\ c_{1}\left(1253\;K\right)=3.704\times 10^{-16} \end{array}$$

From Equations (14) and (17), it can be concluded that the factor *c*_1_ increases exponentially with the rise of temperature. Hence, it can be described as a function of temperature with the following form:

(18) $$c_{1}\left(T\right)=A\;T^{b},\;\;953\;K\leq T<1253\;K$$

The parameters *A* and *b* in Equation (18) were determined using (17). The derived continuous function of *c*_1_ is

(19) $$c_{1}\left(T\right)=\left\{\begin{array}{c} 2.641\times 10^{-86},\;\;\;\;\;\;T<953\;K\\ e^{\left(-4245+590.2\ln\left(T\right)\right)},\;\;\;\;\;\;953\;K\leq T<1253\;K \end{array}\right.$$

**Figure 11:**
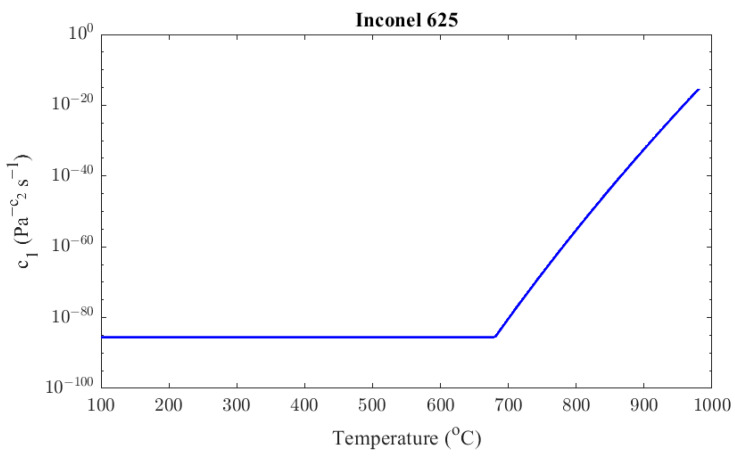
Inconel 625 factor *c*_1_ as a function of temperature.

Figure 11 presents the value of the *c*_1_ parameter across different temperatures.

To conclude, from Equations (9), (16), and (19), the constructed creep model of the Inconel 625 material is

(20) $${\dot{\varepsilon }}_{cr}=\left\{\begin{array}{c} 2.641\times 10^{-86}\;\sigma^{10.84}e^{-\frac{33000}{T}},\;\;\;\;\;\;T<953\;K\\ e^{\left(-4245+590.2\ln\left(T\right)\right)}\;\sigma^{-0.028T+37.47}e^{-\frac{33000}{T}},\;\;\;\;\;\;953\;K\leq T<1253\;K \end{array}\right.$$

**Figure 12:**
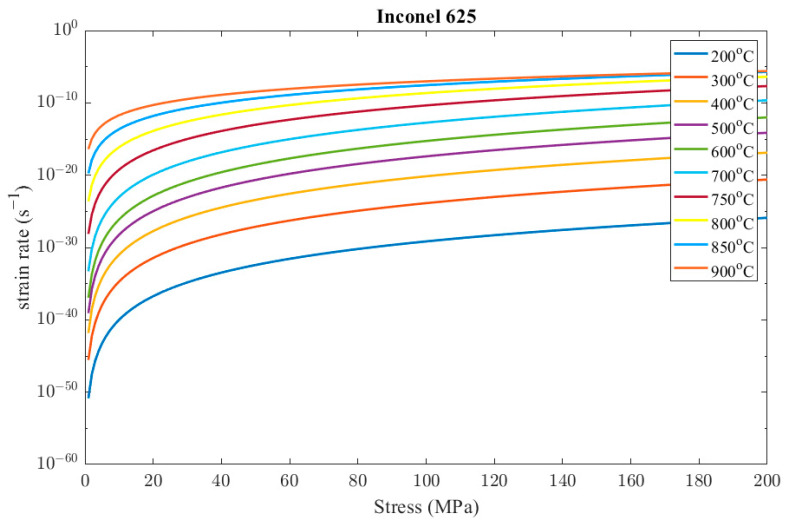
Indicative results of Inconel 625 creep model.

Figure 12 presents the resulting strain rates of the creep model in Equation (20) in the range of temperatures and stresses that occur during the steady-state operation of the recuperator.

**Figure 13:**
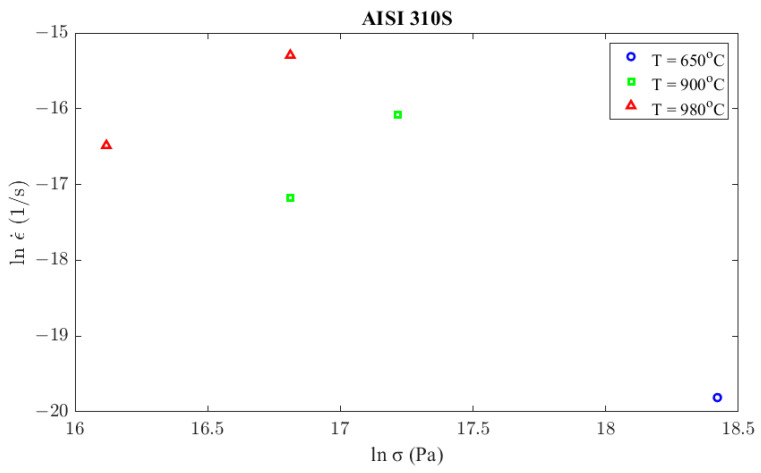
Secondary creep strain rate, calculated from creep experiments according to stress and temperature conditions.

Concerning the creep model that describes the corresponding phenomenon of the AISI 310S material, this was constructed similarly to Inconel 625. Creep tests were conducted at 650 °C, 900 °C, and 980 °C similarly to Inconel 625 but for lower levels of stress since steel is more prone to creep. All the results from the creep tests are presented in Figure 13 and in Table 4.

The critical temperature of AISI 310S is

(21) $$\frac{T_{m}}{2}\approx 740\;{}^{\circ}C$$

The stress exponent, $c_{2}(T)$, can be determined above 740 °C at 900 °C and 980 °C, similarly to the aforementioned case of Inconel 625

(22) $$\begin{array}{c} c_{2}\left(1173\;K\right)=2.71\\ c_{2}\left(1253\;K\right)=1.72 \end{array}$$

**Figure 14:**
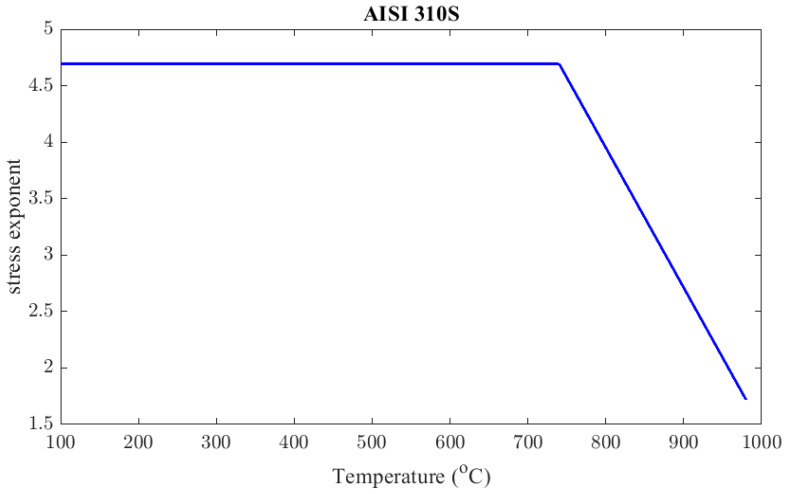
AISI 310S stress exponent, *c*_2_, as a function of temperature.

A typical value of *c*_2_ at temperatures a little lower than the critical one and below 130 MPa is 4.5 [36]. From the results in Equation (22), it is obvious that the value of the stress exponent decreases as temperature increases above the critical one. The fitted linear function is given in Equation (23). The value of $c_{2}$ at the critical temperature from the fitted curve is estimated to be 4.694. Since this value is very close to the reference value, 4.5, and the function has to be continuous, it will be considered that the stress exponent has a value of 4.694 at every temperature below critical. Figure 14 illustrates $c_{2}$ as a function of temperature.

(23) $$c_{2}\left(T\right)=\left\{\begin{array}{c} 4.694,\;\;\;\;\;\;T<1023\;K\\ -0.0124\;T+17.25,\;\;\;\;\;\;1023K\leq T<1253\;K \end{array}\right.$$

Contin et al. [36] demonstrated that the activation energy of AISI 310S is 337 kJ/mol. Therefore, the parameter $c_{3}$ is calculated by Equation (24).

(24) $$c_{3}=\frac{Q_{c}}{R}=40,534\;K$$

The *c*_1_ parameter can be determined from experimental data at 900 °C and 980 °C.

(25) $$\begin{array}{c} c_{1}\left(1173\;K\right)=5.75\times 10^{-13}\\ c_{1}\left(1253\;K\right)=7.27\;{\times 10}^{-6} \end{array}$$

For the AISI 310S material, the $c_{1}(T)$ parameter is described as a function of temperature according to Equation (18), similarly to Inconel 625. The resulting function is

(26) $$c_{1}\left(T\right)=\left\{\begin{array}{c} 9.43\times \;10^{-29},\;\;\;\;\;\;T<1023\;K\\ e^{\left(-1780+248\ln\left(T\right)\right)},\;\;\;\;\;\;1023\;K\leq T<1253\;K \end{array}\right.$$

**Figure 15:**
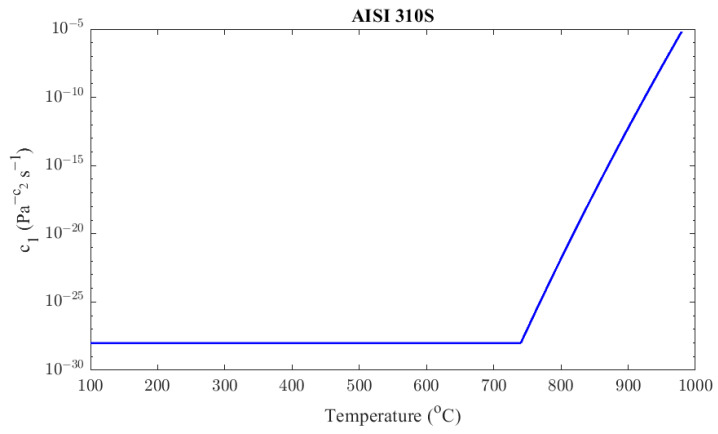
AISI 310S factor *c*_1_ as a function of temperature.

It is considered that the value of the $c_{1}$ coefficient is $9.43\;{\times 10}^{-29}\;{Pa}^{-4.694}s^{-1}$ below 740 °C so that the function does not show any discontinuities. The derived value of $c_{1}$ at 650 °C from the experimental material data is presented in Equation (27), which is very close to the used value (i.e., $9.43\times \;10^{-29}\;{Pa}^{-4.694}s^{-1}$). Figure 15 shows graphically the value of the parameter $c_{1}$ as a function of temperature.

(27) $$c_{1}\left(923K\;\right)=1.17\times 10^{-28}\;{Pa}^{-4.8}s^{-1}\;for\;T=923\;K<T_{m}/2$$

To conclude, the constructed creep model of the AISI 310S material is

(28) $${\dot{\varepsilon }}_{cr}=\left\{\begin{array}{c} 9.43\times 10^{-29}\;\sigma^{4.694}e^{-\frac{40534}{T}},\;\;\;\;\;\;T<953\;K\\ e^{\left(-1780+248\ln\left(T\right)\right)}\;\sigma^{-0.0124\;T+17.25}e^{-\frac{40534}{T}},\;\;\;\;\;\;953\;K\leq T<1253\;K \end{array}\right.$$

**Figure 16:**
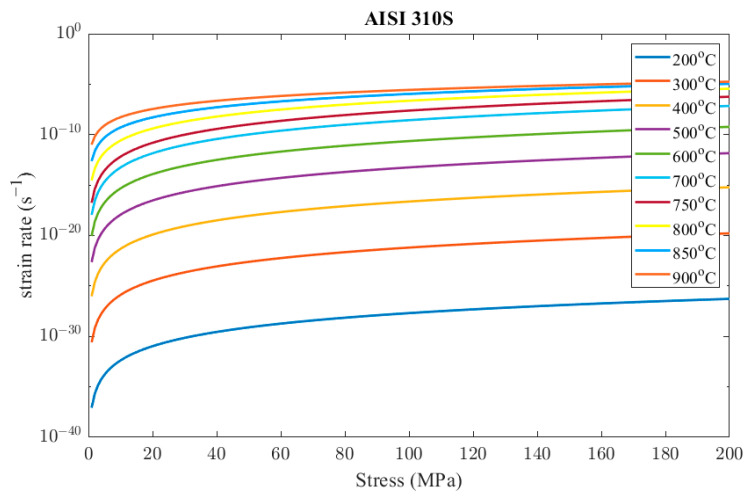
Indicative results of AISI 310S creep model.

Figure 16 presents the resulting strain rates of the creep model in Equation (28) in the range of temperatures and stresses that occur during the steady-state operation of the recuperator.

It should be noted that the creep parameters of the materials will be determined through creep tests and subsequently imported into the FEA model to simulate the response of the recuperator. It is assumed that these parameters will not change significantly due to other failure mechanisms, such as thermal fatigue and oxidation.

## 3. Results

### 3.1. Static Structural

**Figure 17:**
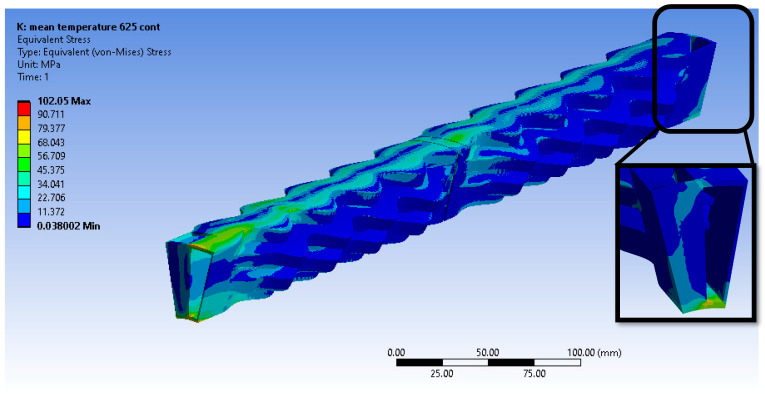
Equivalent von Misses stresses from the tetrahedral mesh—Inconel 625 continuous.

In this paragraph, the results of the static structural simulations for every material are presented. Firstly, the Inconel 625 continuous material results are presented. Figure 17 demonstrates the equivalent von Misses stress field using the presented tetrahedral mesh. It is observed that the material is deformed elastically since its yield point is higher than the emerging stresses at every temperature. However, von Misses stresses up to 71 MPa emerge at an area where the temperature is 875 °C, making this area most prone to creep. As no stress concentration is observed from the supports, it can be concluded that they are properly positioned.

For the determination of the shear force at the roller support and the spring constant at the elastic radial support, the thermal expansion coefficient of the material of the recuperator ($a_{1}$) is taken to be $a_{1}=12.8\;{\times \;10}^{-6}{\;C}^{-1}$ for the Inconel 625 materials and $a_{1}=14.5{\;\times 10}^{-6}{\;C}^{-1}$ for the AISI 310S materials from the ANSYS database. In the case of the continuous Inconel 625, the resulting shear force has a direction towards the center and is equal to 1050 N.

**Figure 18:**
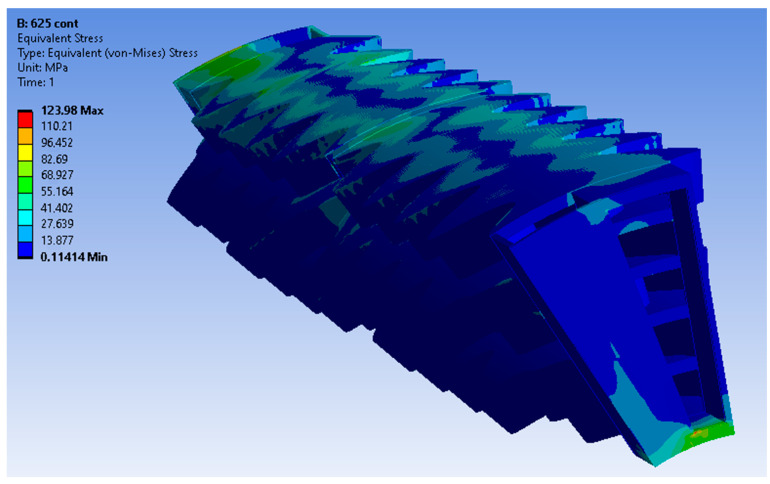
Equivalent von Misses stresses from the hexahedral mesh—Inconel 625 continuous.

The emerging stress field from an FEA model with exactly the same set-up using the hexahedral dominant mesh instead of the tetrahedral one is presented in Figure 18. Even though the maximum stress is given as 124 MPa, it represents an artifact due to an ill-conditioned element in a complex geometrical area of the model. In Figure 17 and Figure 18, it can be observed that the resulting stress fields from the tetrahedral and hexahedral meshes are similar, validating that the results are mesh-independent.

**Figure 19:**
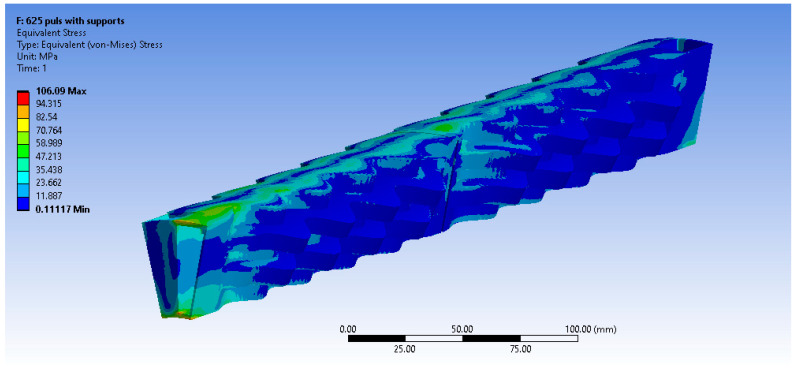
Equivalent von Misses stresses (MPa)—Inconel 625 pulsed.

**Figure 20:**
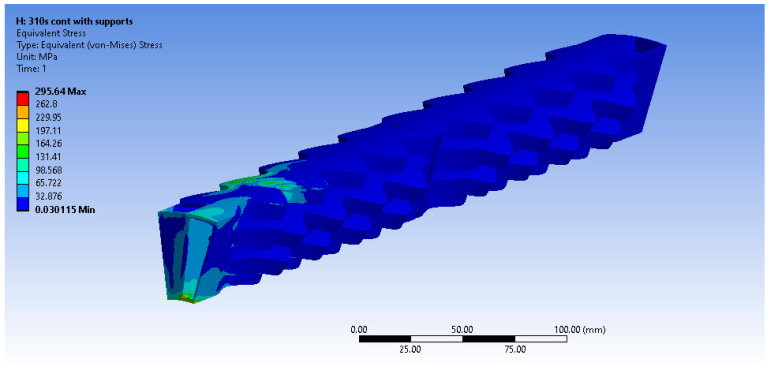
Equivalent von Misses stresses (MPa)—AISI 310S continuous.

**Figure 21:**
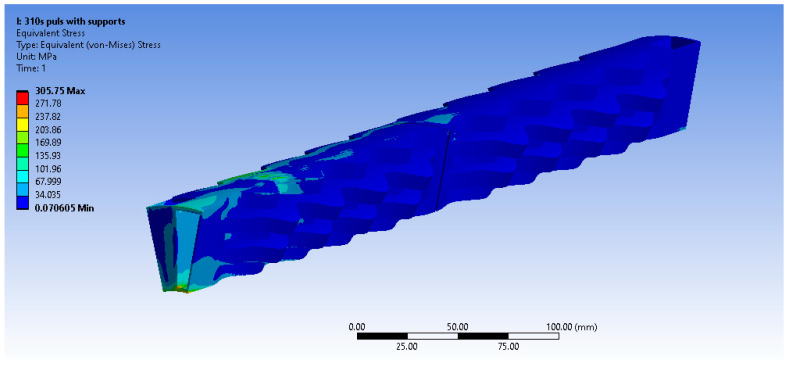
Equivalent von Misses stresses (MPa)—AISI 310S pulsed.

The equivalent von Misses stress fields are presented below (Figure 19, Figure 20 and Figure 21) for the other three materials. It can be observed that in the case of the AISI 310S materials, high stresses emerge close to the supports. This occurs due to the higher thermal expansion coefficient of AISI 310S, which results in the use of a shear force up to 4500 N and a stiffness constant up to $35\;N/{mm}^{3}$. The emerging stresses in the recuperator fabricated from AISI 310S are up to 300 MPa due to its higher thermal expansion coefficient.

### 3.2. Creep and Stress Relaxation

#### 3.2.1. Inconel 625 Results

**Figure 22:**
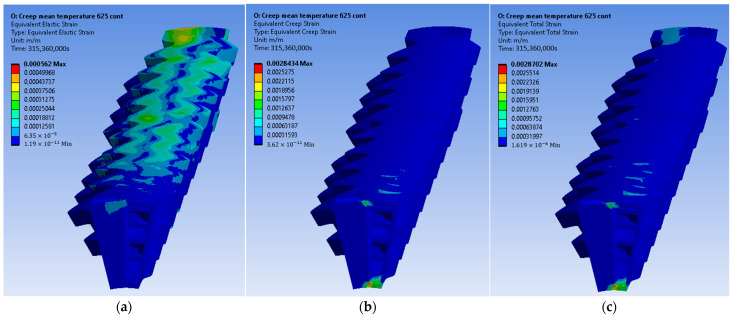
Inconel 625: (**a**) elastic strain, (**b**) creep strain, and (**c**) total strain.

**Figure 23:**
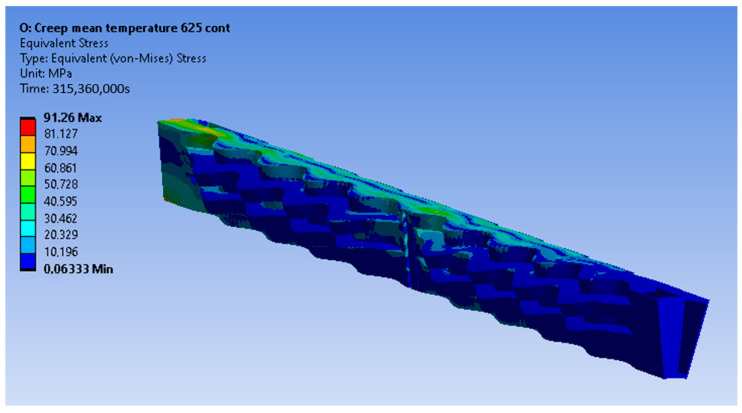
Equivalent stress distribution of the recuperator after the stress relaxation for Inconel 625.

**Figure 24:**
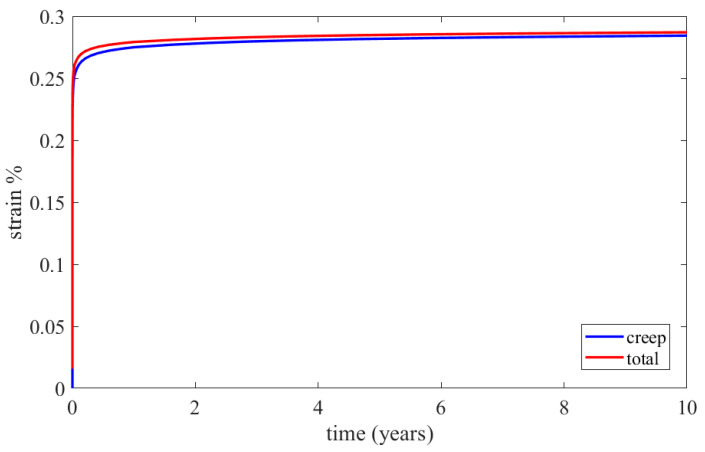
Calculated creep and total equivalent strains for the element with the maximum creep strain rate through the FEA simulation for Inconel 625.

**Figure 25:**
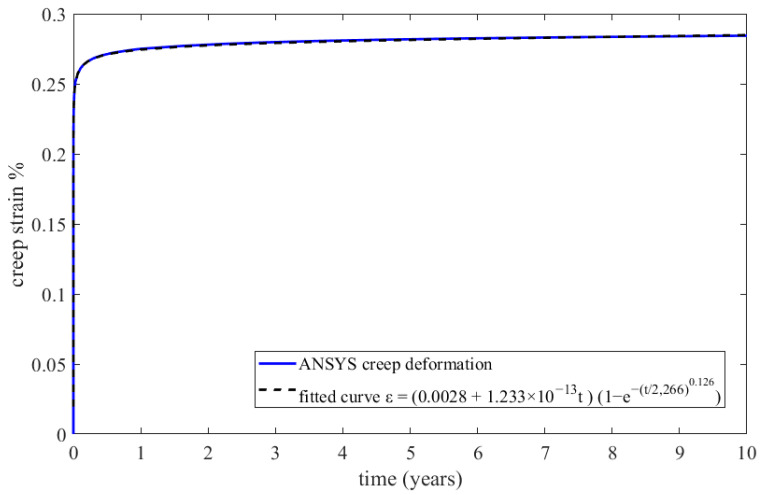
FEA maximum creep strain for Inconel 625 and fitted creep curve.

The constructed creep models for Inconel 625 and AISI 310S, which were described in the previous paragraph, were incorporated into the simulated material properties in the ANSYS Workbench. The simulation time was ten years, and the creep parameters were applied to the simulation. These data proved to be sufficient to identify the thermomechanical response of the recuperator and make predictions about its service life. Firstly, the creep and stress relaxation responses of the recuperator are presented for the Inconel 625 case after ten years of simulation time. The equivalent elastic, creep, and total strains are shown in Figure 22. The region that is affected more by the creep and stress relaxation is the hot end of the recuperator, where the creep deformation is higher and the stress relaxation is more significant (Figure 22b and Figure 23). Figure 24 depicts the creep and total strain calculated by the FEA model as a function of time for the element where creep is more dominant. A function with the form of Formula (29) was fitted to the maximum creep strain curve obtained from the FEA simulation (Figure 25) using genetic algorithms in MATLAB (MathWorks, Natick, Massachusetts, USA). Hence, an analytical expression of the creep strain was acquired (Equation (30)).

(29) $$\varepsilon_{cr}\left(t\right)=\left(A+\lambda t\right)\left(1-e^{-{\left(\frac{t}{T}\right)}^{n}}\right)$$

(30) $$\varepsilon_{cr}\left(t\right)=(0.0028+1.233\times 10^{-13}t)\left(1-e^{-{\left(\frac{t}{2.266\times 10^{3}}\right)}^{0.126}}\right)$$

**Figure 26:**
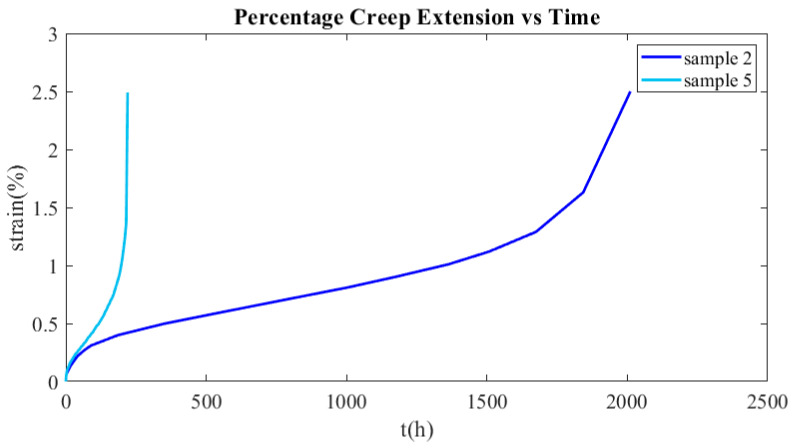
Experimental creep curves of samples 2 and 5.

**Figure 27:**
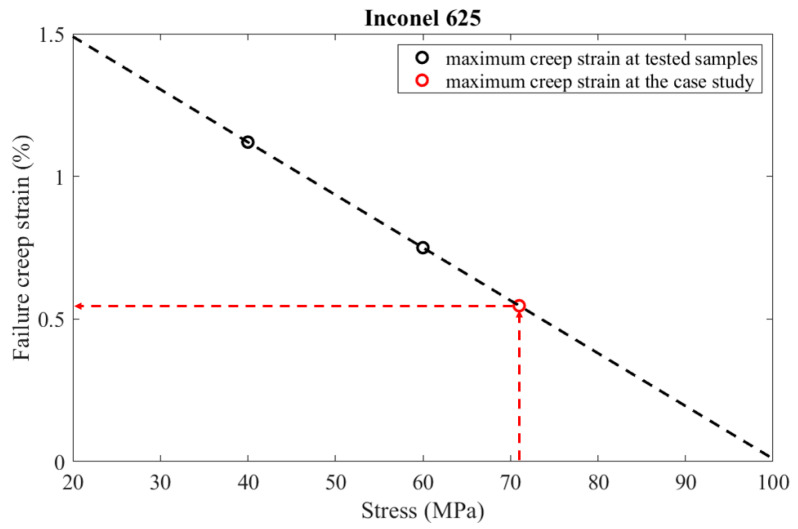
Estimation of the creep strain, where the tertiary creep begins at the area of the recuperator where creep is more dominant.

In order to determine an appropriate failure criterion, experimental data are used from two specimens tested at temperatures close to the operating one. The tertiary creep phase of sample 2, which was tested at 900 °C and 60 MPa, started at a creep strain of 1.12%, while the tertiary creep phase of sample 5, which was tested at 900 °C and 40 MPa, started at a creep strain of 0.75%. The aforementioned values of the critical creep strain were obtained from the creep curves presented in Figure 26. In the specific case study, the maximum creep deformation occurs in the hot region of the recuperator, where the temperature is 875 °C and the emerging stress is 71 MPa. Hence, the operating temperature is close to 900 °C, at which the creep tests were performed. Since the emerging stress is 71 MPa, the diagram presented in Figure 27 was constructed in order to estimate the capacity of creep strain at that level of stress, assuming a linear relationship between failure creep strain and stress level. As the dashed lines illustrate in Figure 27, the maximum creep strain is 0.55%. However, the time when the strain reaches 0.5% was considered a failure criterion in order not to have excessive creep deformation. Hence, the lifespan is calculated by Equation (30) and is equal to 566 years, which is certainly much higher than the burner service life.

#### 3.2.2. AISI 310S Results

**Figure 28:**
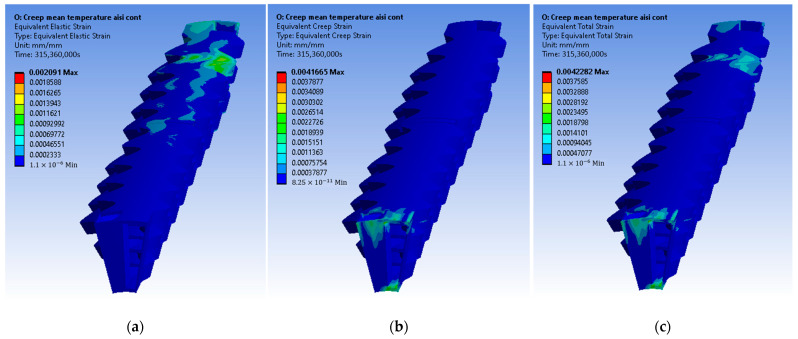
AISI 310S: (**a**) elastic strain, (**b**) creep strain, and (**c**) total strain.

**Figure 29:**
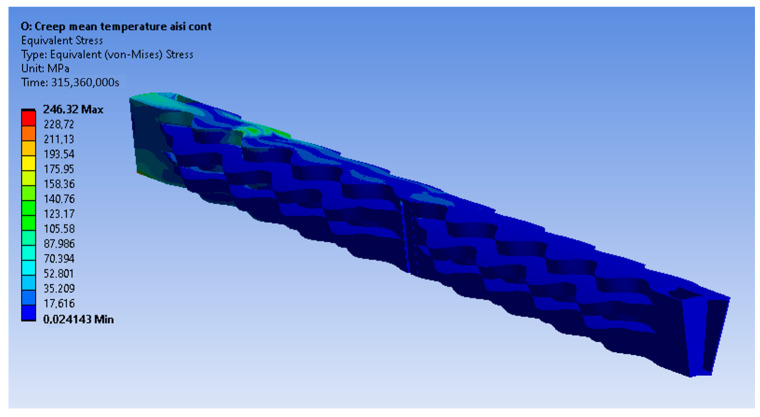
Equivalent stress distribution of the recuperator after the stress relaxation for AISI 310S.

**Figure 30:**
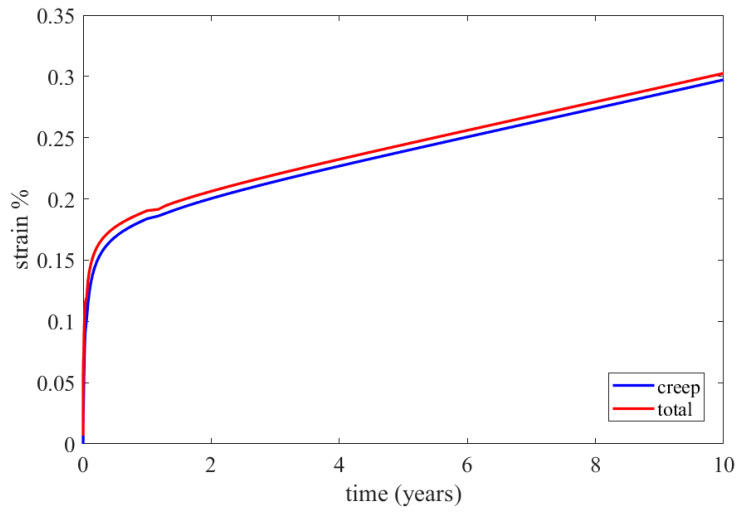
Calculated creep and total equivalent strains for the element with the maximum creep strain rate by the FEA simulation for AISI 310S.

The results of the creep and stress relaxation simulation with AISI 310S are presented in the following figures. Figure 28 and Figure 29 present the equivalent strain and stress fields after ten years of simulation. The equivalent, creep, and total strains are presented as a function of time in Figure 30 for the element with the maximum creep strain rate.

**Figure 31:**
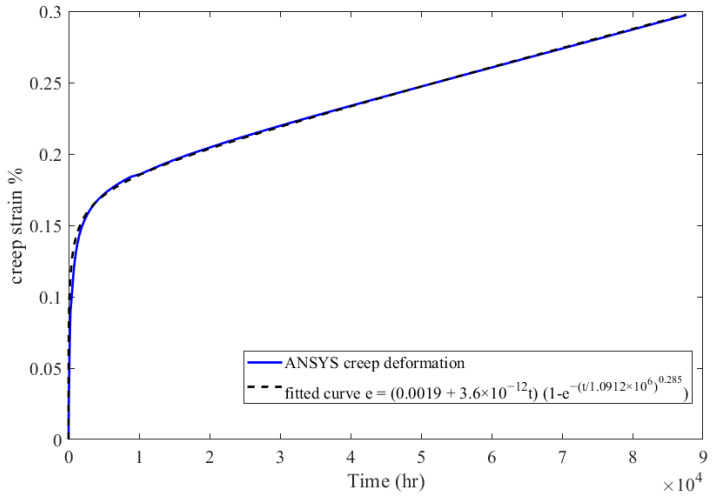
FEA maximum creep strain for AISI 310S and fitted creep curve.

In order to estimate when the recuperator will fail due to the excessive creep deformation, an extrapolation of the simulation results should be conducted. Formula (29) was fitted to the curve of the maximum equivalent creep that was obtained from the FEA simulation. The best fit of the parameters of the function was accomplished with the use of genetic algorithms. The fitted function is presented in Equation (31) and illustrated in Figure 31, along with the FEA results.

(31) $$\varepsilon_{cr}\left(t\right)=\left(0.0019+t\;3.6\times 10^{-12}\right)\left(1-e^{-{\left(\frac{t}{1.09\times 10^{6}}\right)}^{0.285}}\right)$$

**Figure 32:**
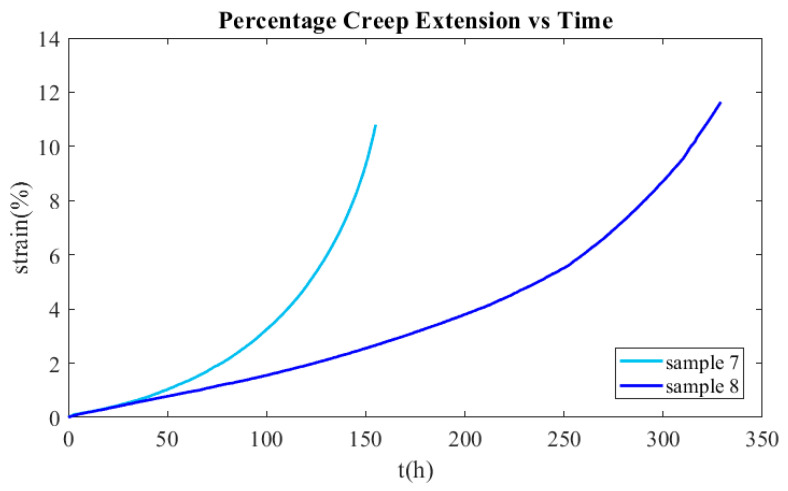
Experimental creep curves of samples 7 and 8.

The creep curves of samples 7 and 8 tested at 900 °C and 30 MPa and 20 MPa, respectively, are presented in Figure 32. It can be observed that the AISI 310S was greatly deformed until the failure. Moreover, the transition from the secondary to the tertiary creep phase is not distinct at all. Hence, the time when the creep strain reaches 0.5% will be considered the failure criterion. Using Equation (31), it is estimated that the lifespan of the recuperator made from AISI 310S is 27 years.

It can be observed that Inconel 625 demonstrates significantly improved creep performance. As indicated by Formula (29), the parameter λ, which is a function of the thermomechanical properties of the material, plays a significant role in influencing its creep performance. This parameter determines the slope of the asymptotic line on the creep curve. In the case of Inconel 625, the parameter λ is one order of magnitude lower in comparison to AISI 310S, while, conversely, its service life is one order of magnitude longer.

## 4. Conclusions

The results of the study show that a recuperator manufactured by AISI 310S will fail due to excessive creep deformation after 27 years of operation, while the one manufactured by Inconel has a service life one order of magnitude longer. The recuperators manufactured by continuous or pulsed technology have negligible differences since their emerging stress distributions are basically the same. The steps followed to reach those conclusions are as follows:

- The mechanical properties of the material were determined using both experimental data from the tensile tests and the literature;
- An FEA model was set up with the appropriate geometry model, boundary conditions, and mesh;
- The stress relaxation/creep deformation material models were constructed using both experimental data from the creep tests and data from the literature;
- Creep and stress relaxation simulations were conducted for every material, with a simulation time of 10 years. This data proved to be sufficient to identify the thermomechanical response of the recuperator and make predictions about its service life;
- The obtained creep strain curves from the FEA simulation were fitted with a function derived by genetic algorithm techniques. Hence, an analytical expression of the creep strain as a function of time was obtained;
- Failure criteria were set based on the experimental data, and the lifespan of the recuperator was estimated.

This study adds to the understanding of the material properties of Inconel 625 and 316 Stainless Steel fabricated using additive manufacturing, since there is a lack of comprehensive data in the existing literature pertaining to their creep properties. Furthermore, this research offers valuable insights into how these materials respond to creep and stress relaxation in an industrial-scale case study, highlighting the reliability of AM-fabricated components for use in industrial applications.

## Figures and Tables

**Table 1:** Chemical composition of the Inconel 625.

Element	Al	Co	Cr	Fe	Mn	Mo	Nb	Ni	P	Si	Ti
wt (%)	0.04	0.02	21.55	3.11	<0.01	9.00	4.00	Bal.	0.006	0.03	0.01

**Table 2:** Chemical composition of the AISI 310S.

Element	Fe	Cr	Ni	Mn	Si	Al
wt (%)	50.77	25.97	19.3	2.03	1.57	0.3

**Table 3:** Creep experimental data of Inconel 625.

	Initial Diameter	Initial Length	Duration (h)	Stress (MPa)	Temperature (°C)	Strain Rate (%/h)
sample 1	5.62	29.80	143	400	650	9.97 × 10^−2^
sample 2	5.62	29.83	220	60	900	3.11 × 10^−3^
sample 3	5.61	29.87	230	30	980	1.14 × 10^−3^
sample 4	5.63	29.90	1547	20	980	4.21 × 10^−4^
sample 5	5.63	29.88	2052	40	900	4.62 × 10^−4^

**Table 4:** Creep experimental data of AISI 310S.

	Initial Diameter	Initial Length	Duration (h)	Stress (MPa)	Temperature (°C)	Strain Rate (%/h)
sample 6	5.62	29.87	2316	100	650	8.961 × 10^−4^
sample 7	5.62	29.89	163	30	900	3.739 × 10^−2^
sample 8	5.61	29.88	384	20	900	1.246 × 10^−2^
sample 9	5.61	29.86	60	20	980	8.205 × 10^−2^
sample 10	5.62	29.89	238	10	980	2.492 × 10^−2^

**Figure A1:**
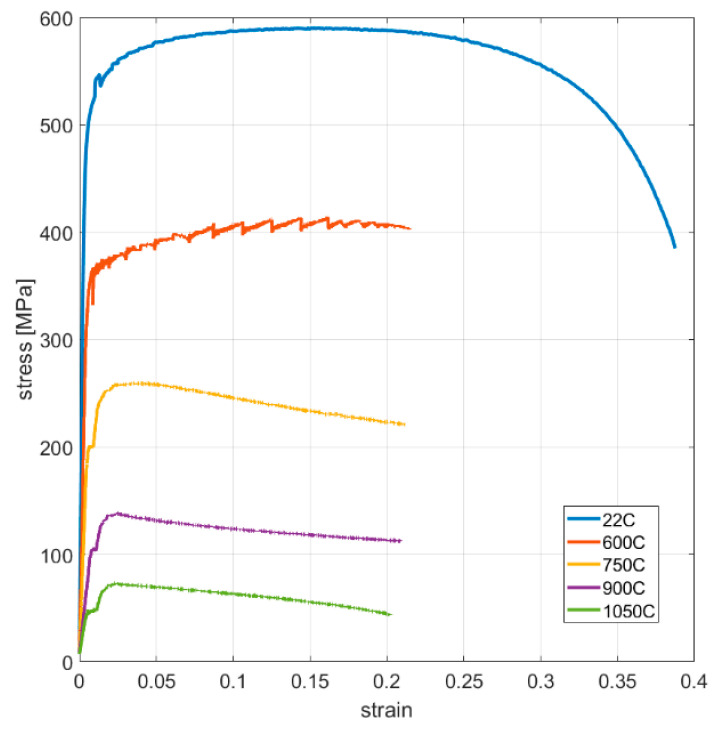
Engineering stress–strain curves of vertically oriented samples.

**Figure A2:**
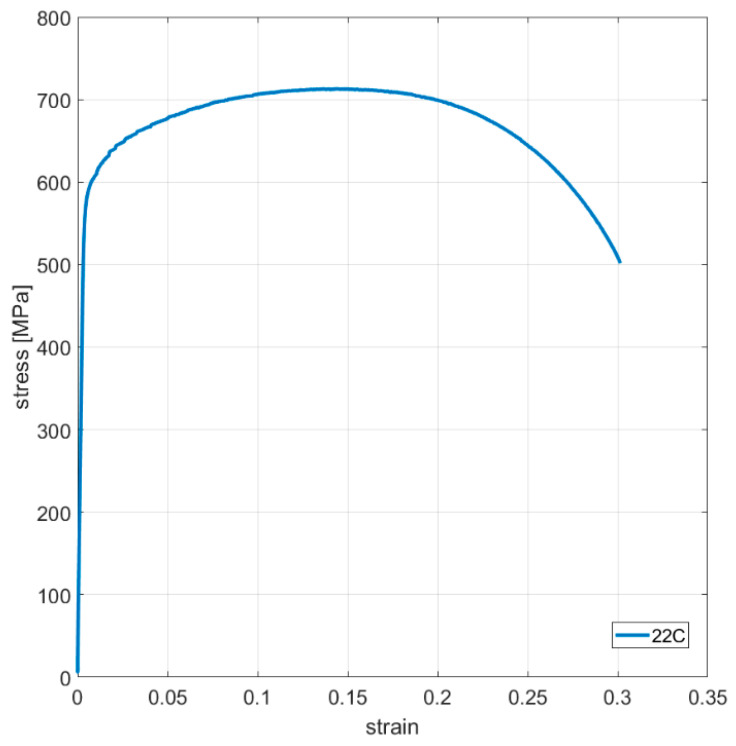
Engineering stress–strain curves of horizontally oriented samples.

**Figure A3:**
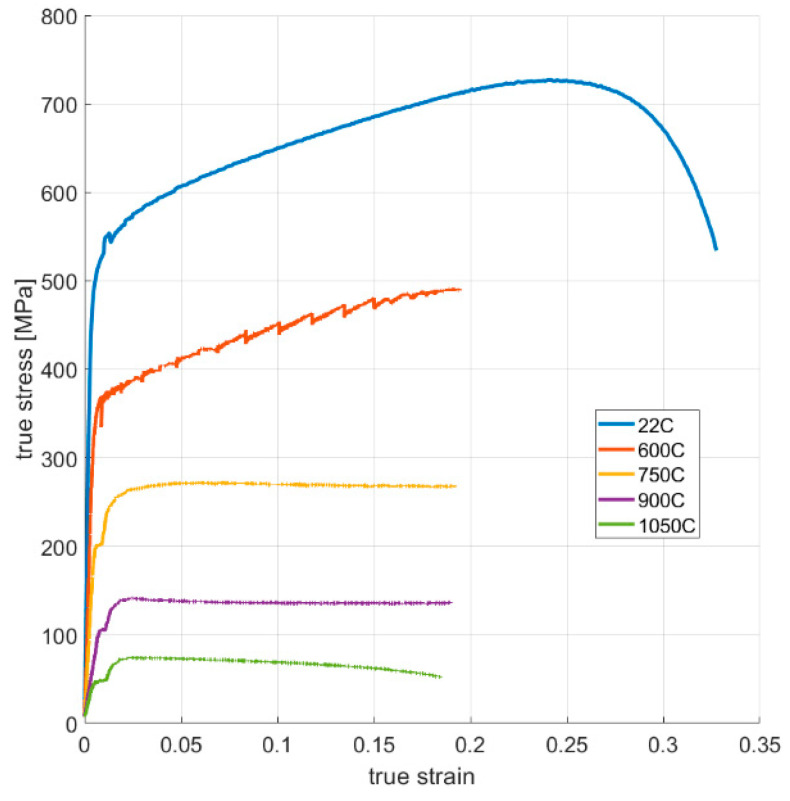
True stress–strain curves of vertically oriented samples.

**Figure A4:**
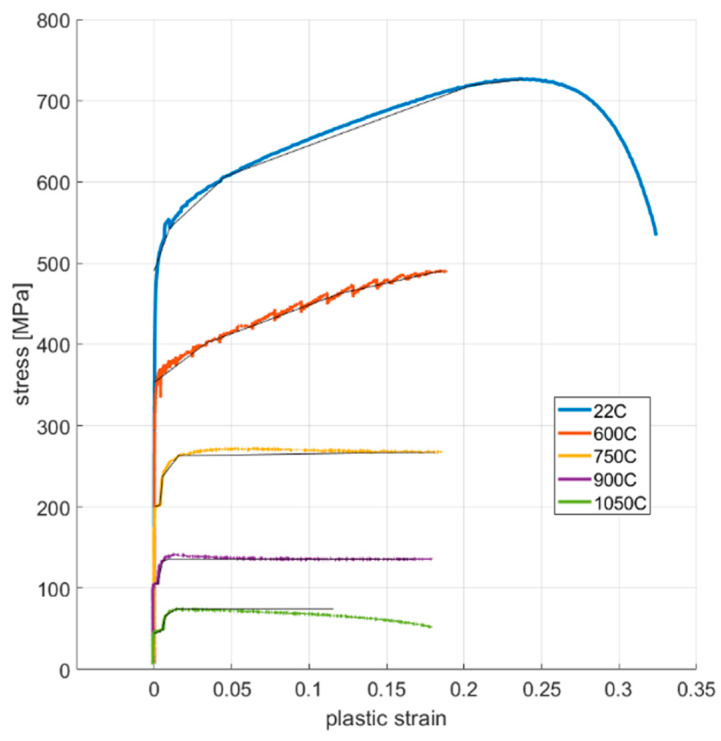
True stress–plastic strain curves and multilinear data (black lines) imported in FEA.

**Figure A5:**
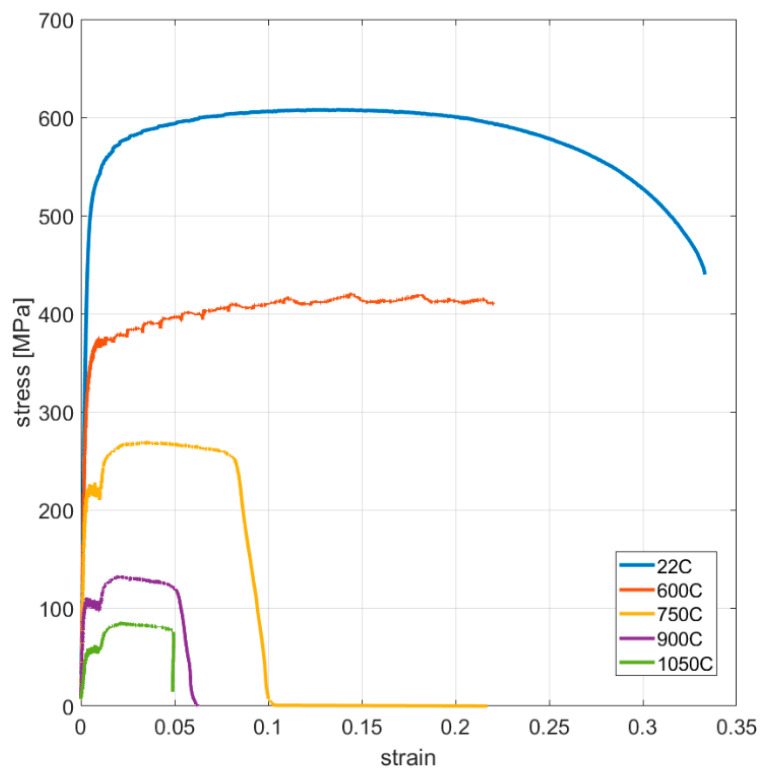
Engineering stress–strain curves of vertically oriented samples.

**Figure A6:**
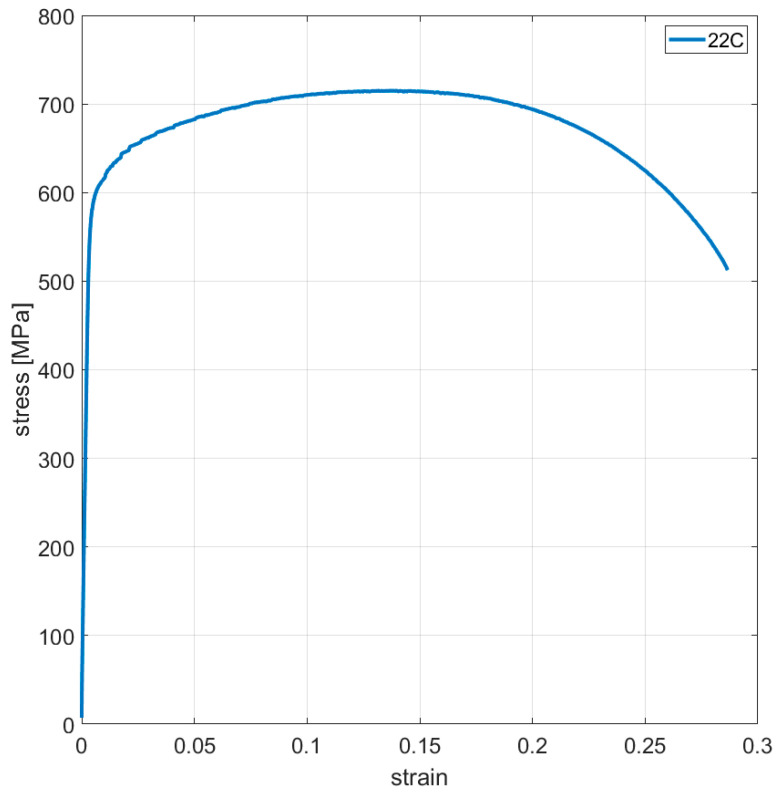
Engineering stress–strain curves of horizontally oriented samples.

**Figure A7:**
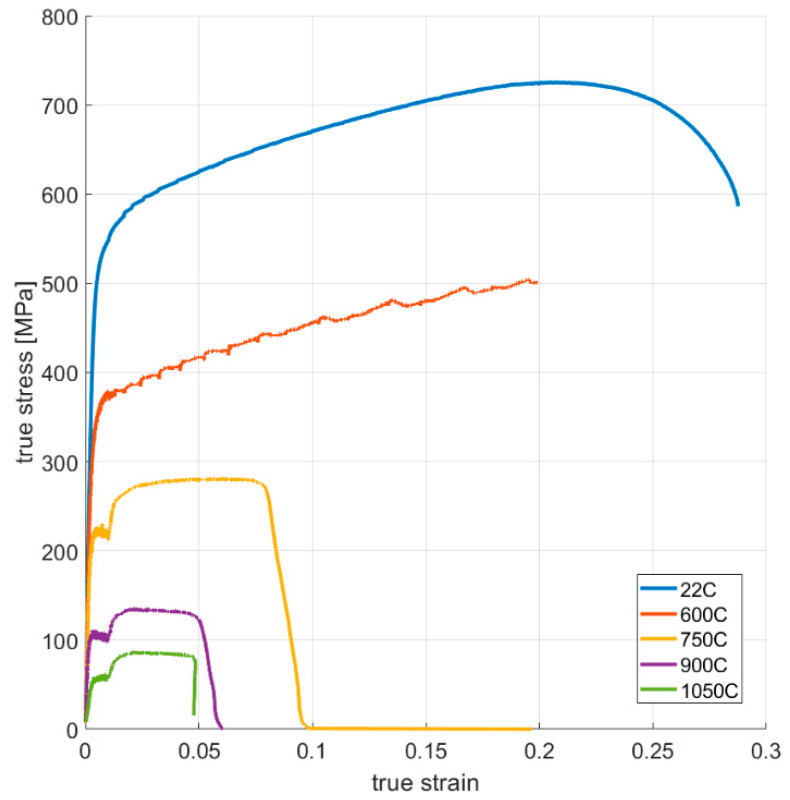
True stress–strain curves of vertically oriented samples.

**Figure A8:**
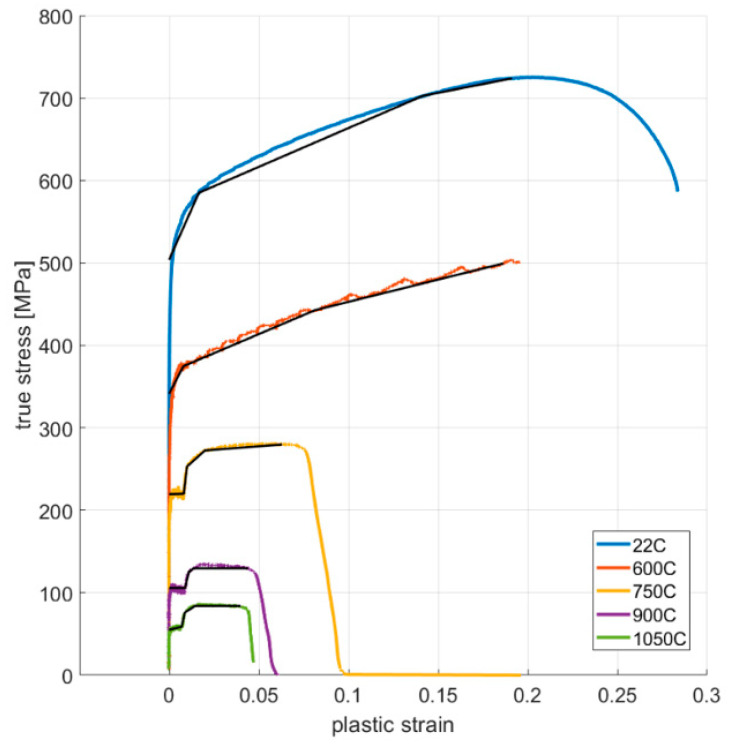
True stress–plastic strain curves and multilinear data (black lines) imported in FEA.

**Figure A9:**
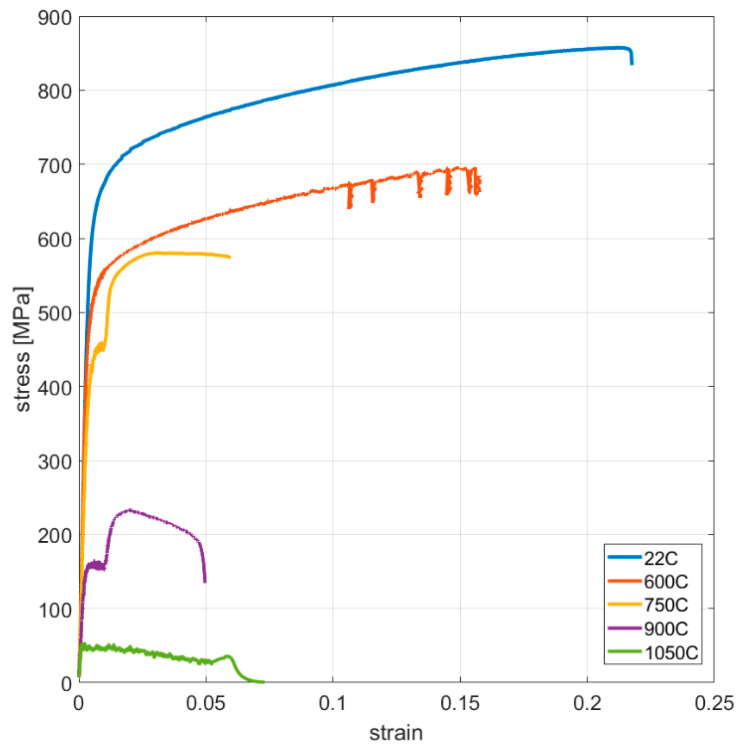
Engineering stress–strain curves of vertically oriented samples.

**Figure A10:**
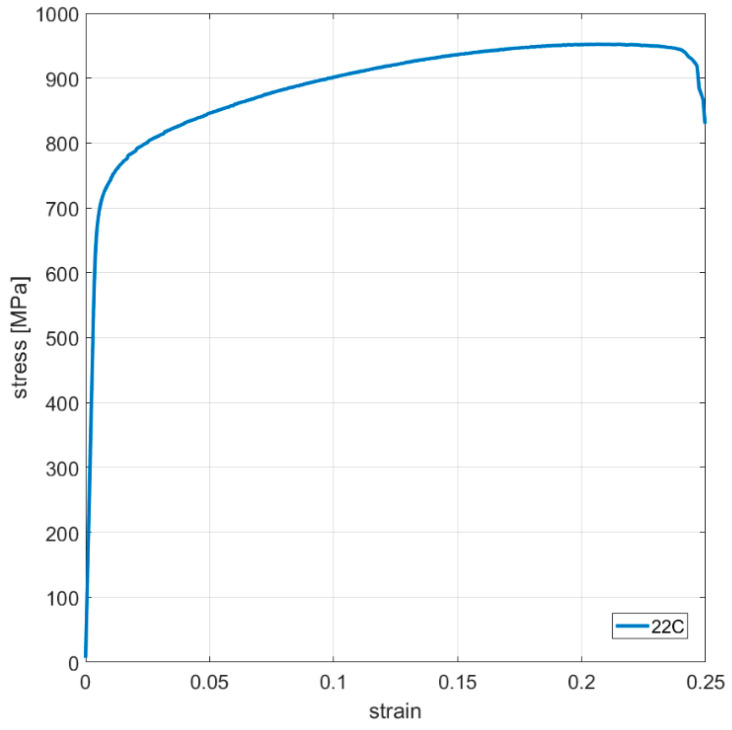
Engineering stress–strain curves of horizontally orientated samples.

**Figure A11:**
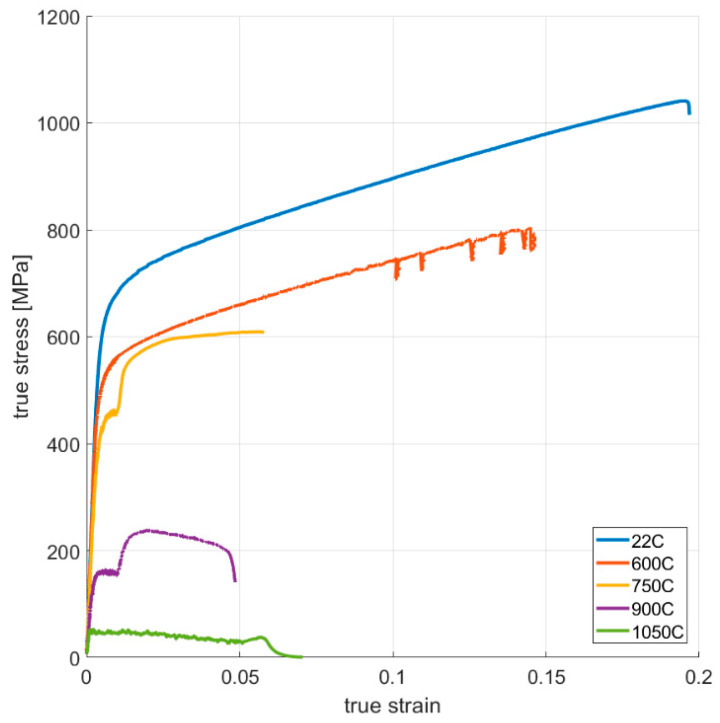
True stress–strain curves of vertically oriented samples.

**Figure A12:**
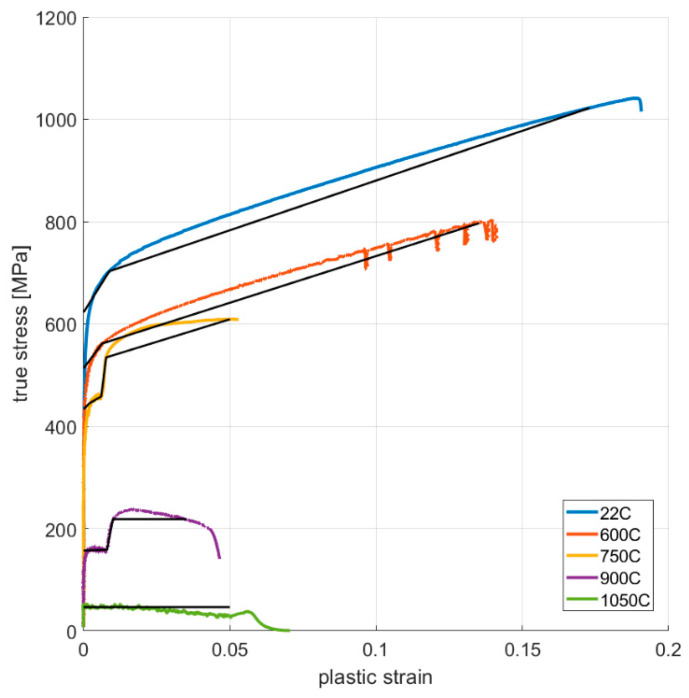
True stress-–strain curves and multilinear data (black lines) imported in FEA.

**Figure A13:**
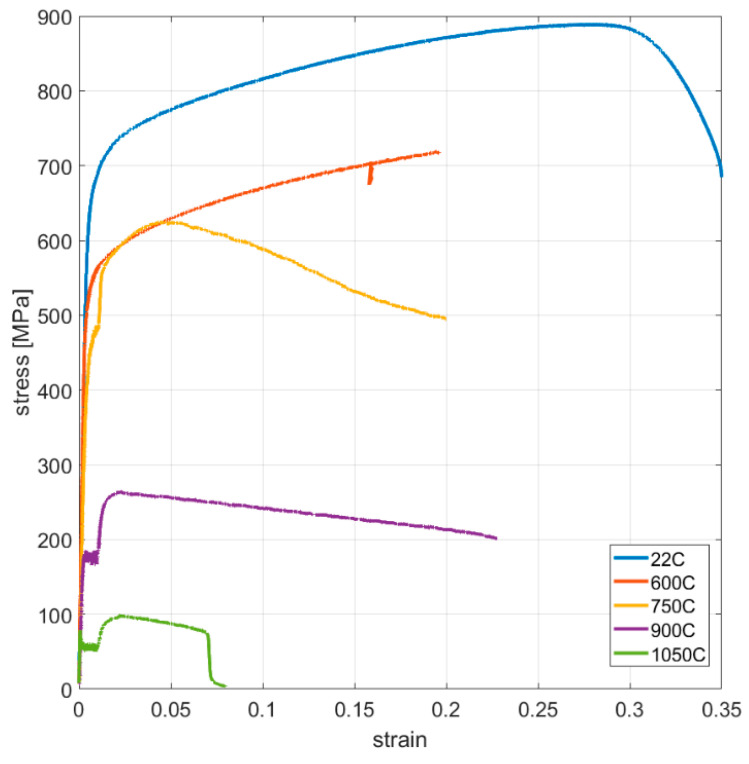
Engineering stress–strain curves of vertically oriented samples.

**Figure A14:**
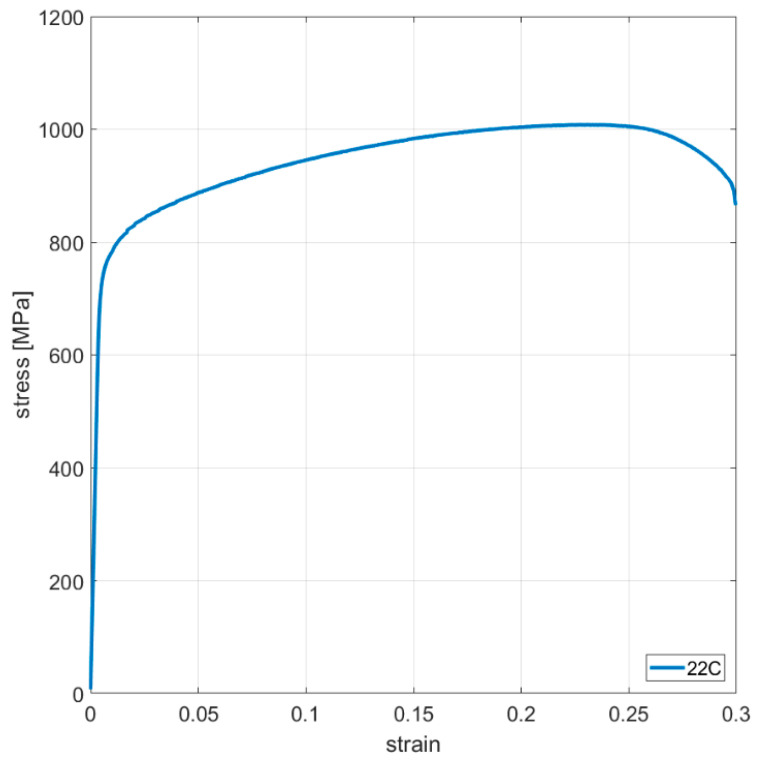
Engineering stress–strain curves of horizontally oriented samples.

**Figure A15:**
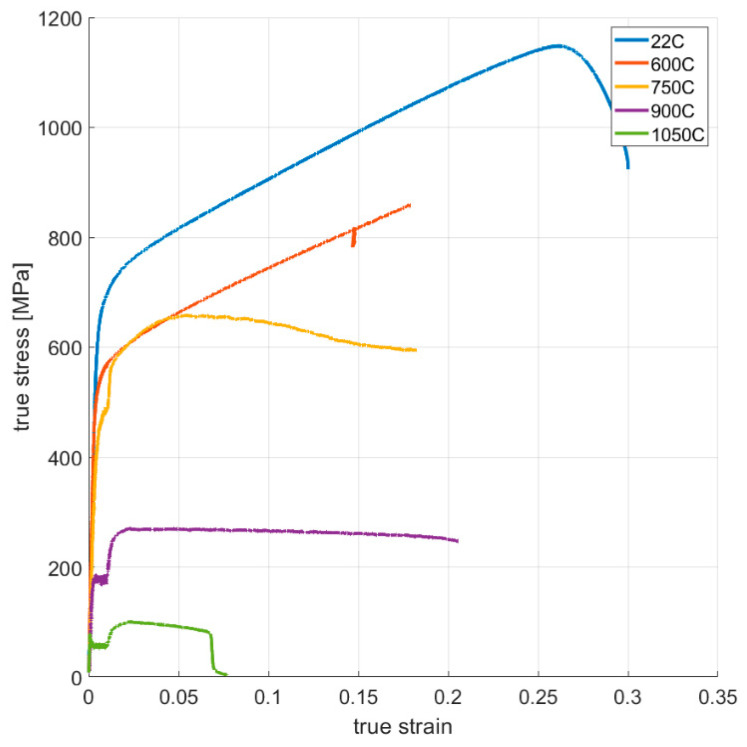
True stress–strain curves of vertically oriented samples.

**Figure A16:**
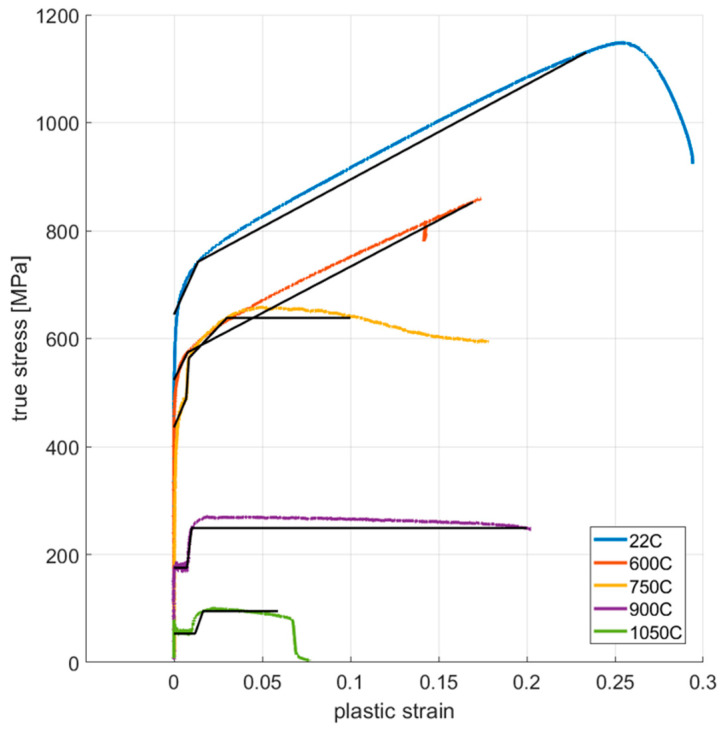
True stress–plastic strain curves and multilinear data (black lines) imported in FEA.

## Data Availability

Data are contained within the article.

## Appendix A. Mechanical Properties

### Appendix A.1. 310 Stainless Steel Continuous Material Model

**Table A1:** AISI 310S Continuous - Orthotropic Elasticity.

Temperature (°C)	Young’s Modulus X Direction (MPa)	Young’s Modulus Y Direction (MPa)	Young’s Modulus Z Direction (MPa)	Poisson’s Ratio XY	Poisson’s Ratio YZ	Poisson’s Ratio XZ	Shear Modulus XY (MPa)	Shear Modulus YZ (MPa)	Shear Modulus XZ (MPa)
20	165,000	165,000	159,800	0.25	0.25	0.25	6.39 × 10^4^	6.6 × 10^4^	6.6 × 10^4^
600	77,400	77,400	75,000	0.32	0.32	0.32	2.84 × 10^4^	2.93 × 10^4^	2.93 × 10^4^
750	40,000	40,000	38,768	0.3	0.3	0.3	1.49 × 10^4^	1.54 × 10^4^	1.54 × 10^4^
900	13,000	13,000	12,563	0.24	0.24	0.24	5.07 × 10^3^	5.24 × 10^3^	5.24 × 10^3^
1050	9090	9090	8803	0.24	0.24	0.24	3.55 × 10^3^	3.67 × 10^3^	3.67 × 10^3^

### Appendix A.2. 310 Stainless Steel Pulsed Material Model

**Table A2:** AISI 310S Pulsed - Orthotropic Elasticity.

Tempe-rature (C)	Young’s Modulus X Direction (MPa)	Young’s Modulus Y Direction (MPa)	Young’s Modulus Z Direction (MPa)	Poisson’s Ratio XY	Poisson’s Ratio YZ	Poisson’s Ratio XZ	Shear Modulus XY (MPa)	Shear Modulus YZ (MPa)	Shear Modulus XZ (MPa)
20	173,789	173,789	151,454	0.25	0.25	0.25	6.06 × 10^4^	6.95 × 10^4^	6.95 × 10^4^
600	150,000	150,000	131,000	0.32	0.32	0.32	4.96 × 10^4^	5.68 × 10^4^	5.68 × 10^4^
750	112,000	112,000	98,000	0.3	0.3	0.3	3.77 × 10^4^	4.31 × 10^4^	4.31 × 10^4^
900	76,600	76,600	66,795.56	0.24	0.24	0.24	2.69 × 10^4^	3.09 × 10^4^	3.09 × 10^4^
1050	21,900	21,900	19,070	0.24	0.24	0.24	7.69 × 10^3^	8.83 × 10^3^	8.83 × 10^3^

### Appendix A.3. Inconel 625 Continuous Material Model

**Table A3:** Inconel 625 Continuous - Orthotropic Elasticity.

Temperature (°C)	Young’s Modulus X Direction (MPa)	Young’s Modulus Y Direction (MPa)	Young’s Modulus Z Direction (MPa)	Poisson’s Ratio XY	Poisson’s Ratio YZ	Poisson’s Ratio XZ	Shear Modulus XY (MPa)	Shear Modulus YZ (MPa)	Shear Modulus XZ (MPa)
22	167,610	167,610	164,259	0.278	0.278	0.278	64,264.08	65,575.12	65,575.12
600	155,333.6	155,333.6	152,228	0.305	0.305	0.305	58,324.9	59,514.77	59,514.77
750	122,872.6	122,872.6	120,416	0.33	0.33	0.33	45,269.17	46,192.7	46,192.7
900	71,734.17	71,734.17	70,300	0.33	0.33	0.33	26,428.57	26,967.73	26,967.73
1050	46,938.43	46,938.43	46,000	0.33	0.33	0.33	17,293.23	17,646.03	17,646.03

### Appendix A.4. Inconel 625 Pulsed Material Model

**Table A4:** Inconel 625 Pulsed - Orthotropic Elasticity.

Temperature (°C)	Young’s Modulus X Direction (MPa)	Young’s Modulus Y Direction (MPa)	Young’s Modulus Z Direction (MPa)	Poisson’s Ratio XY	Poisson’s Ratio YZ	Poisson’s Ratio XZ	Shear Modulus XY (MPa)	Shear Modulus YZ (MPa)	Shear Modulus XZ (MPa)
20	178,750.8	178,750.8	152,202.7	0.278	0.278	0.278	5.95 × 10^4^	6.99 × 10^4^	6.99 × 10^4^
600	182,907.5	182,907.5	155,742	0.305	0.305	0.305	5.97 × 10^4^	7.01 × 10^4^	7.01 × 10^4^
750	161,146.6	161,146.6	137,213	0.33	0.33	0.33	5.16 × 10^4^	6.06 × 10^4^	6.06 × 10^4^
900	88,081.98	88,081.98	75,000	0.33	0.33	0.33	2.82 × 10^4^	3.31 × 10^4^	3.31 × 10^4^
1050	149551.5	149,551.5	127,340	0.33	0.33	0.33	4.79 × 10^4^	5.62 × 10^4^	5.62 × 10^4^
